# Facile fabrication of ternary MWCNTs/ZnO/Chitosan nanocomposite for enhanced photocatalytic degradation of methylene blue and antibacterial activity

**DOI:** 10.1038/s41598-022-09571-5

**Published:** 2022-04-08

**Authors:** Mitra Malekkiani, Abbas Heshmati Jannat Magham, Fatemeh Ravari, Mehdi Dadmehr

**Affiliations:** 1 Department of Chemistry, Payame Noor University, Tehran, Iran; 2 Department of Biology, Payame Noor University, Tehran, Iran

**Keywords:** Biochemistry, Biological techniques, Environmental sciences, Chemistry, Materials science, Nanoscience and technology

## Abstract

Developing a cheap, stable and effective photocatalyst is necessary for remediation of persistent organic pollutants. To address this challenge, we proposed a unique interfacial engineering technique and proper bandgap matching strategy to synthesize MWCNTs/ZnO/Chitosan ternary nanocomposite for effective photocatalytic application. The features of the prepared samples were determined by FESEM, TEM, EDX, elemental mapping, AFM, FT-IR, XRD, UV–Vis spectroscopy and BET surface analysis. The obtained results showed successful fabrication of synthesized nanocomposites with enhanced surface area. Degradation effect of nanostructures on methylene blue (MB) and antibacterial activity against *Escherichia coli* (*E. coli*), *Staphylococcus aureus* (*S. aureus*) and *Bacillus subtilis* (*B. subtilis*) pathogenic strains were investigated. The proposed photocatalytic mechanism illustrated the electron transfer facilitated by MWCNTs/ZnO/Chitosan structure which results in spatial separation of electron–hole pairs. Compared with ZnO and ZnO/Chitosan, the prepared MWCNTs/ZnO/Chitosan ternary nanocomposite showed high usage of UV illumination and superior separation of photogenerated electron–hole pairs. MWCNTs/ZnO/Chitosan illustrated 86.26% adsorption rate and outstanding increased photocatalytic activity on MB degradation efficiency of 98.76% after 20 min. Stability of photocatalyst reached from 98.76% initial decolorization to 85% at the fourth cycle. In addition, the ternary nanocomposite also exhibited remarkable bactericidal activity against gram-positive (*S. aureus*) and (*B. subtilis*) and gram-negative (*E. coli*) bacteria strains. Due to the obtained results, the prepared nanocomposite would be an efficient candidate photocatalyst with antibacterial properties.

## Introduction

Development of industrial activities and the growth of the world population has caused serious environmental problems such as water pollution and subsequently led to limited access to clean water throughout the world^1^. Due to the occurrence of such problems in recent years, scientists have made several attempts toward removal of existed pollutants such as toxic organic, chemicals substances and pathogenic microorganisms in water reservoirs^2^. A lot of industries, including the textile, tanneries and other chemical factories, continuously release a great amount of polluted wastewater originated from harmful waste materials like dyes, heavy metals and surfactants, which are major health issues all around the world^3,4^.

Some dyes, especially those use in the textile industry, show great resistance against microbiological degradation and some of them have carcinogenesis and mutagenesis properties which are regarded as the major health risk factors for human beings and all other living organisms in the world^5,6^. One of the most commonly used dyes in industries, medicine and chemical activities is Methylene Blue (MB) which is a heterocyclic organic compound with sulfonic group and N≡N bonds in its molecular structure. Water contamination with MB causes serious health problems such as blindness, respiratory disorders and digestive diseases^7^. In addition, the presence of MB in water, even at extremely low concentrations impacts photosynthesis due to reducing the sunlight penetration. So, refining processes for the MB dye molecule removal from industrial wastewaters is crucial before being released into the environment^8^. Besides the dyes, the existence of pathogens and pollutants microorganisms in water encourages the researchers to develop new technologies to control infections and contagious diseases caused by pathogenic bacteria. These attempts resulted in introducing effective approaches against pathogenic microorganism propagation and growth^2,9^.

There are several chemical and physical methods to refine dye-containing industrial wastewaters, which include adsorption, electrochemical precipitation, membrane separation processes, chemical precipitation, photocatalysis, coagulation flocculation, ozonation, biological methods, membrane filtration^10,11^ and sono-oxidation. Most of these treatments are not effective in the refining of dye contaminated effluents because most of them solely transferring the pollutants from one phase to another one without more processing and therefore lead to secondary pollution^12^.

Advanced oxidation processing (AOP) which is based on multiply structures photocatalysts has been applied as an alternative method for treatment of contaminated water samples^11^. These approaches are based on platforms with lower cost, nontoxic features, facile operation, higher efficiency and nondestructive properties^13^. Most of the AOP strategies generate hydroxyl radicals that cause the oxidation process and following mineralize organic and inorganic contaminants in water^11^. The photocatalysis mediated with semiconductors has been regarded recently to overcome environmental challenges like removing pollutants from wastewater^10,14^. Excitation of electrons in the valence band of semiconductors by ultraviolet or visible light results in electron transfer to the conduction band. This phenomenon leads to the production of electron–hole pairs, that in turn generates some free radicals which are involving in oxidation–reduction reactions. So far, many photocatalysts such as Fe_2_O_3_, ZnO, TiO_2_, Bi_2_O_3_, CdS, ZnS and WO_3_ have been used for biological and industrial applications^15,16^. Among them, ZnO has a critical and useful broad bandgap of 3.37 eV with essential binding energy (60 meV). Moreover, ZnO in heterogeneous photocatalysis has been extensively applied for treating wastewater due to its distinguished photocatalytic property, abundance in nature, low price, high chemical stability, suitable optical band gap, low toxicity and environmental resistance. It has been demonstrated that ZnO showed higher photocatalytic performance than common TiO_2_ photocatalyst^17^. Despite mentioned advantages, ZnO photocatalyst still exhibits low photocatalytic activity due to various problems like low photon utilization efficiency, the high recombination rate of charge carriers and narrow spectrum range with a wide bandgap, etc. So, more treatment of ZnO is required to improve its photocatalytic efficiency^7,18^.

There are diverse methods to address above mentioned challenges, such as the development of novel photocatalysts with the constituents with wider bandgaps, and also metal nanoparticles as an electron sink, or stabilizing semiconductor catalysts on some materials which have much larger surfaces. The usage of stable catalysts in the photocatalyst structure has economic advantages on a large scale due to the separation and reusability of the catalyst after the process^19^. The development of hybrid composites has dramatically increased since they showed a wide range of usage, which involves water treatment, antimicrobial properties and photocatalysis^20,21^. Chitosan is a heteropolymer which composed of glucosamine and acetyl glucosamine units. The application of Chitosan and heterogeneous oxide materials is increasing because of the unique properties of its major compounds and synergistic impacts on the final product. In recent years, Chitosan is greatly used in different sections of biosensors for medical applications, as a nanoparticle protecting layer. It exhibited distinguished properties such as nontoxicity, biodegradability, biocompatibility, and high absorbance ability. Many kinds of research have depicted that Chitosan can considerably increase photocatalytic activity while integrated with a composite catalyst because of its high potential in the adsorption of different organic dyes^22^. ZnO/Chitosan nanocomposite has extensively attracted vast interest due to its unique applications, like UV protection and antimicrobial activity in recent years^23^.

The interaction of contaminant with Chitosan/metallic oxide photocatalyst happens through chelation, coordination of NH_2_ groups, co-precipitation and ligand exchange or electrostatic interaction with protonated NH_2_ groups. Adsorption of various sorts of contaminants is attributed to the unprotonated NH_2_ group of the Chitosan as the ligand. So, protonation reduces the pollutant adsorption and the best performance of photocatalyst would be at higher pH values. Even dispersion of catalyst in water is another challenge for applied catalysts which must be addressed in the construction of photocatalysts. The modification of the Chitosan by means of physical and chemical methods can alter its physicochemical properties and further impact its adsorption behavior to overcome the limitation of a particular photocatalyst^24^. An efficient method for improving the physic/chemical properties of Chitosan is its combination with inorganic fillers, which contain Silica, hydroxyapatite, Calcium phosphate cements and clay nanoparticles that are frequently use to sustain the Chitosan matrix^25^.

Carbon nanotubes (CNTs) are cylindrical nanomaterials that exhibit remarkable electrical conductivity, thermal conductivity and mechanical features. The modification of CNTs with Chitosan has improved the treatment of mechanical and thermal properties compared to pure Chitosan, which may extend the application of Chitosan^25^. Recently, Multi-Wall Carbon nanotubes (MWCNTs) have been extensively used as catalytic material because of their unique properties. It has been demonstrated that combining ZnO with carbonaceous nanomaterials like CNTs is more efficient than the ZnO photocatalyst^19,26^. The reason is the inhibition effect of CNT on recombination rate between generated electron and hole pairs through accepting electrons from ZnO^11^. In addition, some features of CNTs, such as good chemical stability and corrosion resistance, suppress the photo corrosion effect of ZnO in CNT/ZnO composite structure and hence improve the photocatalytic efficiency^23^. It was shown that functionalization of CNTs by ZnO nanoparticles increase the photocatalyst activity via enhancing the adsorption of light by photocatalyst, improving the transport charge and increasing the surface area, which consequently inhibits the recombination of electron–hole pairs^19^. So, it would be possible to enhance the efficiency of photocatalysts through the construction of nanocomposites by incorporating MWCNTs, ZnO and Chitosan.

**Figure 1:**
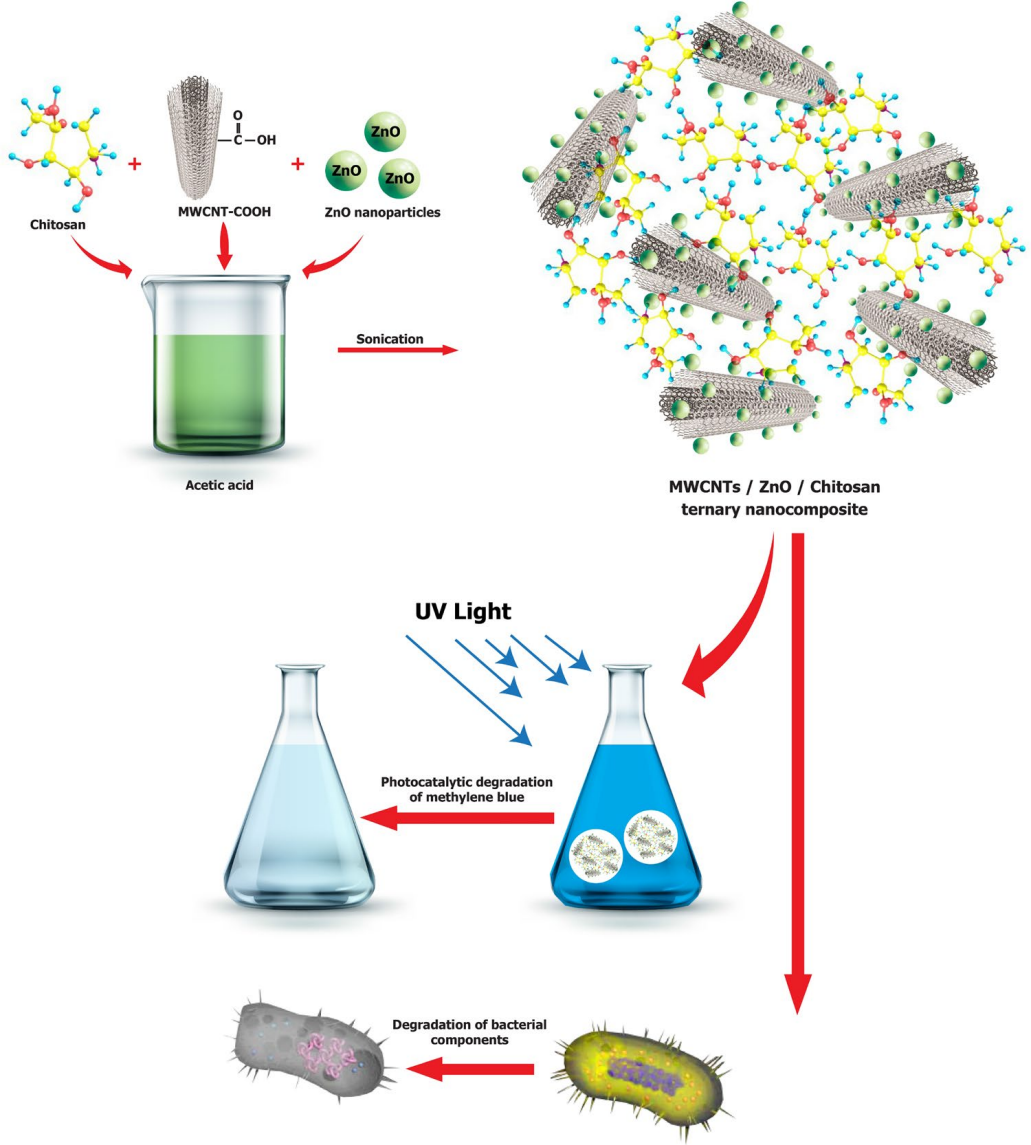
Schematic illustration of the synthesis and photocatalytic activity of MWCNTs/ZnO/Chitosan ternary nanocomposite.

In the present research, the ZnO nanoparticle, ZnO/Chitosan nanocomposite and MWCNTs/ZnO/Chitosan ternary nanocomposite have been synthesized to determine their photocatalytic activities (Fig. 1). Morphological and properties of synthesized nanocomposite photocatalysts were characterized by FT-IR, UV–Vis, XRD, SEM, EDAX, TEM, AFM and BET analysis. Then, photocatalytic degradation and adsorption of MB as the target dye has been investigated in the presence of UV light irradiation. The antibacterial effects of photocatalysts were also surveyed on gram-negative (*Escherichia coli*) and gram-positive bacteria (*Staphylococcus aureus* and *Bacillus subtilis*). The performance of photocatalysts was studied through optimization of parameters including catalyst weight, dye concentration, pH of the solution and the temperature on the MB degradation. To the best of our knowledge, this is the first report of MWCNTs/ZnO/Chitosan nanocomposites used for photocatalytic degradation and adsorption of MB pollutants under UV light irradiation.

## Experimental

### Chemicals and materials

Deionized water was used for the preparation of the solution throughout the experiment. MWCNTs with diameters of 10–40 nm and lengths of 1–25 µm were obtained from the Research Institute of Iran’s Petroleum Industry. Low molecular weight Chitosan was purchased from Sigma-Aldrich, Munich, Germany. The chemicals which were applied in this research included zinc sulfate heptahydrate (ZnSO_4_·7H_2_O, 99%), polyethylene glycol (PEG), ammonium hydroxide (NH_4_OH), acetic acid (CH_3_COOH, 99%), sodium hydroxide (NaOH, 98%), nitric acid (HNO_3_, 70%), hydrochloric acid (HCl, 37%) and Methylene blue (MB) were obtained from Merck (Germany). The bacteria strains, including *Bacillus subtilis*, *Staphylococcus aureus* and *Escherichia coli*, were prepared from the Department of Microbiology, University of Mashhad, Iran. The medium of Nutrient broth was purchased from Quelab, Canada. All chemicals used were of analytical grade and were used as received without any further purification.

### Synthesis of ZnO nanoparticles

In this experiment, zinc sulfate heptahydrate (ZnSO_4_·7H_2_O) was used as a precursor to prepare ZnO nanoparticles. The sol–gel method was applied in the present study. Briefly, 5 g of polyethylene glycol dissolved in 10 ml of deionized distilled water while the mixture stirring continuously. Then, 2.875 g ZnSO_4_·7H_2_O was added to the above solution, and the mixture stirring was kept constantly at room temperature for 30 min till the homogenous solution was obtained. Then, 0.1 M NH_4_OH was used in order to titrate the above solution to make a pH 7 and the prepared mixture solution was magnetically stirred for another 1 h at 60 °C to complete the dehydration process of Zn(OH)_2_ suspension and thick colloidal white sediment was formed. Then, the obtained solution cooled down to room temperature. The final product was collected by centrifugation at 9000 rpm and washed several times with water and once with ethanol. This precipitation dried for 12 h at 100 °C and then calcinated in air conditions at 500 °C for 2 h to prepare ZnO nanoparticles.

### Synthesis of ZnO/Chitosan nanocomposite

To prepare ZnO/Chitosan nanocomposite, firstly, 1 g of ZnO nanoparticles was dissolved in 100 ml of 1% (v/v) acetic acid. After that, to the above solution 750 mg of low molecular weight Chitosan was added. The mixture was sonicated for 20 min, and then 1 M NaOH was added dropwise till the mixture was adjusted at the pH 10. Later on, the mixture solution was heated in the water bath for 3 h at 65 °C. Ultimately, the precipitate was collected using centrifugation and washed with deionized water several times, and at last, it was dried in an oven at 50 °C for 1 h^27^.

### Functionalization of MWCNTs

In a typical reaction, 2 g of MWCNTs heated up at 350 °C for 30 min to separate amorphous carbon. After heating, 0.5 g of MWCNTs were oxidized with 60 ml of condensed nitric acid solution at 100 °C for 12 h. The mixture was filtered and washed precisely with de-ionized water several times after cooling down the temperature to room heat. Then the pH of the final wash reached neutral and following under vacuum conditions. The precipitate was dried to obtain carboxylic carbon nanotubes (MWCNTs-COOH)^28^.

### Synthesis of MWCNTs/ZnO/Chitosan

For the preparation of MWCNTs/ZnO/Chitosan, 0.2% Chitosan solution with pH 5 was obtained by solving appropriate amount of Chitosan flakes in 0.1 mol l^−1^ acetic acid and then stirred magnetically at room temperature at 3 h to dissolve completely. The suitable amount of pre-prepared ZnO nanoparticles and MWCNTs were dispersed in 0.2% Chitosan mixture solution, so the final mass ratio of MWCNTs/ZnO/Chitosan was 3:1:100. The solution was then sonicated after stirring 6 h for 20 min. At last, a high dispersed black colloidal solution was obtained and dried at the environment temperature^29^.

### Apparatuses

Adsorption spectra and photocatalytic degradation were determined through Ultraviolet–Visible (UV–Vis) spectrophotometer (Shimadzu, double beam, Japan) along with quartz cells and also a single beam spectrometer (Jenway, UK) was employed to optimize the photocatalytic degradation conditions. To stir the appointed solution a magnetic stirrer and a heater were used to perform the photocatalyst test under UV-A lamp Philips 11 W illumination. High energy sonicator (Hielscher ultrasonic processor 200 W, 50 kHz, UTR 200, Germany) with 10 mm of titanium was used for homogenization of the solution.

### Characterization techniques

Morphological and physical characteristics of synthesized nanoparticles and nanocomposites were determined by different characterization methods. By using an X-ray diffraction device (XRD, Philips analytical diffractometer), the X-ray diffraction models were attained through determination of crystalline nature of nanoarchitecture. FT-IR spectroscopy was done by employing a Fourier transform infrared spectroscopy (FT-IR, Shimadzu-8400, Japan). UV–Vis spectrophotometer (Shimadzu, Japan) was used to determine the optical bandgaps of prepared photocatalysts. Morphology and sizes of synthesized nanomaterials were identified by transmission electron microscope (TEM, Aleo 912 AB) and field emission scanning electron microscopy (FESEM energy-dispersive) as well as X-ray spectroscopy (EDX Tescan Brno-Mira Lmu). The surface morphology was also characterized using atomic force microscopy (AFM, model Full plus), in tapping mode. Bet belsorp mini II instrument was used to determine the BET surface area of applied nanomaterials by physical adsorption of nitrogen at 100 °C and 300 °C, the samples were degassed for 4 h before experiments.

### Photocatalytic degradation analysis

In order to assess the photocatalytic performance of as-prepared photocatalysts, the degradation of MB by irradiation of UV light was assayed. In this study, to accomplish the best conditions, the degradation time, temperature impact, pH effect, dose concentration, and dye concentration factors were investigated to determine the optimum condition in the photocatalytic degradation process. After achieving this optimization, 25 mg of the photocatalyst were added to 100 ml of 5 mg l^−1^ aqueous solution of MB under the specified temperature of 313 K and pH 9. This suspension was stirred magnetically in darkness for 60 min to receive equilibrium of adsorption/desorption. Then, the photocatalytic process was started under UV irradiation. A 11 W UV-lamp was employed as the light source, so that the distance between the base of the beaker under UV light was calculated to be 20 cm. The experiment was conducted under UV light for 300 min while every 20 min, 5 ml of sample was taken and following centrifuged to remove the particles of catalyst for analysis. The photocatalytic estimation activity is based upon the MB photo-adsorption at the maximum absorption wavelength of 664 nm using UV–Vis spectroscopy, the intensity of each sample was recorded over time. The MB degradation percentage has been estimated from Eq. (1)^30^.

1 $$Dye \,degradation\, \left({\%}\right)=\frac{{C}_{0}-{C}_{t}}{{\text{C}}_{t}} \times 100,$$

where C_0_ and C_t_ in this equation show the initial concentration after the equilibrium of adsorption–desorption for 20 min and the real-time concentration of MB, respectively.

### Adsorption tests

Similarly, the adsorption capability of photocatalysts was investigated by the adsorption tests of MB in the dark conditions. 25 mg of photocatalyst were added to 100 ml of the aqueous solution of MB with the same concentration under the special temperature of 313 K and pH 9 and then stirred for 60 min in darkness to establish the equilibrium of absorption–desorption. Following this, 5 ml of samples were removed at a regular 30 min time intervals and photocatalysts were taken out utilizing centrifugation. The samples were analyzed by UV–Vis spectrophotometry at the wavelength (λ_max_) 664 nm after centrifuging.

### Antibacterial assays

Disk diffusion procedure was applied for the investigation of the antibacterial function of applied nanomaterials. The *Escherichia coli*, *Staphylococcus aureus* and *Bacillus Subtilis* strains were grown in the Nutrient Broth (NB) broth medium in the shaker incubator at 37 °C. The bacteria samples (50 µl) were dispersed on the NB plates. The filter paper discs that are sterilized were inserted on the surface of inoculated plates. Then, 15 µl of the nanomaterial solutions were pipetted on the disk and following incubated overnight at 37 °C. The minimum inhibitory concentration was achieved by using measuring of growth inhibition zone induced by the released compounds of nanostructures^3,31^.

## Results and discussion

### Morphology and structural characteristics of prepared nanostructures

**Figure 2:**
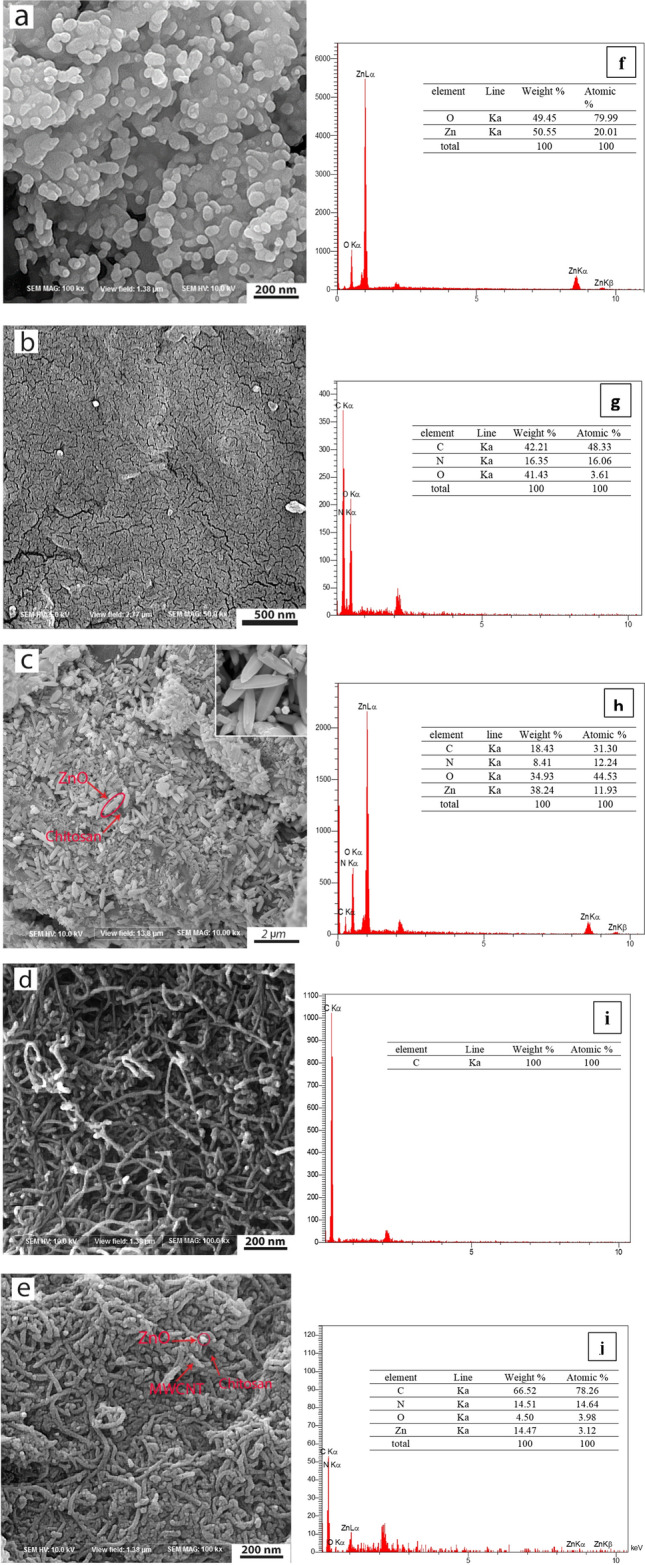
FESEM images of (**a**) pure ZnO, (**b**) pure Chitosan, (**c**) ZnO/Chitosan nanocomposite, (**d**) pure MWCNTs and (**e**) MWCNTs/ZnO/Chitosan nanocomposite; EDS spectra of (**f**) pure ZnO, (**g**) pure Chitosan, (**h**) ZnO/Chitosan nanocomposite, (**i**) pure MWCNTs and (**j**) MWCNTs/ZnO/Chitosan nanocomposite.

Morphology is an important property for the evaluation of catalyst effectiveness. The morphology and structure of the synthesized nanoparticles were analyzed by FESEM, TEM and AFM microscopes. Figure 2 shows the FESEM images of ZnO nanoparticles, Chitosan, MWCNTs, ZnO/Chitosan and MWCNTs/ZnO/Chitosan nanocomposites. As illustrated in Fig. 2a, ZnO nanoparticles had almost a uniform spherical shape with some agglomeration. It was also apparent that the sample was naturally porous, which was in accordance with previously published manuscripts^19^. Additionally, it was determined that the ZnO nanoparticles possess a mean diameter around 21.63 nm (Fig. S1a). The FESEM image in Fig. 2c revealed a uniform distribution of ZnO nanoparticles with small agglomeration on the Chitosan surface^32^. As illustrated in the inset of Fig. 2c, ultrasonic mediated synthesis results in the transformation of spherical ZnO NPs to the hexagonal structure. A similar modification of ZnO nanosheets to the hexagonal ZnO nanorods also has been reported in the same sonication-based synthesis strategy^33^. As shown in Fig. 2c and Fig. S1b, the ZnO/Chitosan nanocomposite with a rod shape, showed a mean size of about 20–80 nm. In accordance with our researches, Bharathi et al. reported that the morphology of the biologically produced Chitosan seeds, which were coated with ZnO nanoparticles had the rod shape grains^34^. As observed in Fig. 2b, the Chitosan surface was smooth. It has been reported that during the formation of nanocomposites, ZnO nanoparticles uniformly dispersed over the Chitosan surface, and the smoothness of the surface was disappeared^35^. As revealed in Fig. 2d, the MWCNTs had an almost tubular structure where the outer diameter was nearly 10–40 nm and the length of the tube was measured about 1–25 µm. Moreover, in Fig. 2e, the exhibited surface of MWCNTs/ZnO/Chitosan showed a uniform coating of MWCNTs on Chitosan, which differs from the pristine Chitosan surface. The observed MWCNTs coating was due to the electrostatic interaction between the negatively charged MWCNTs and the positively charged polycation of Chitosan. The interaction reduces the Van der Waals forces in MWCNTs bundles because of wrapping of the MWCNTs in polymer chains. The functionalized MWCNTs includes polar functional groups on their surface which facilitate the accumulation of nanoparticles and results to preparation of nanocomposites with average diameter about 28.83 nm (Fig. S1c). Additionally, the uniform and small sizes of the catalyst particles contribute not only to higher adsorption of dye on the nanocomposite surface but also to more light absorption by photocatalytic system.

To investigate the composition of ZnO/Chitosan and MWCNTs/ZnO/Chitosan nanocomposites, the energy-dispersive X-ray spectroscopy (EDS) was employed. The percentage of the elements present in the prepared nanocomposite is shown in the table in Fig. 2h. The spectrum revealed a peak of oxygen and zinc, which shows the presence of ZnO nanoparticles. The other observed peaks of nitrogen and carbon in the EDS spectrum were assumed to arise from the amine (–NH_2_) group of Chitosan^34^. Figure 2j represented, the main elements of Zn, O, N and C in this spectrum which showed that MWCNTs are placed on Chitosan and ZnO nanoparticles are attached to MWCNTs and no other impurity was detected in samples by this analysis.

**Figure 3:**
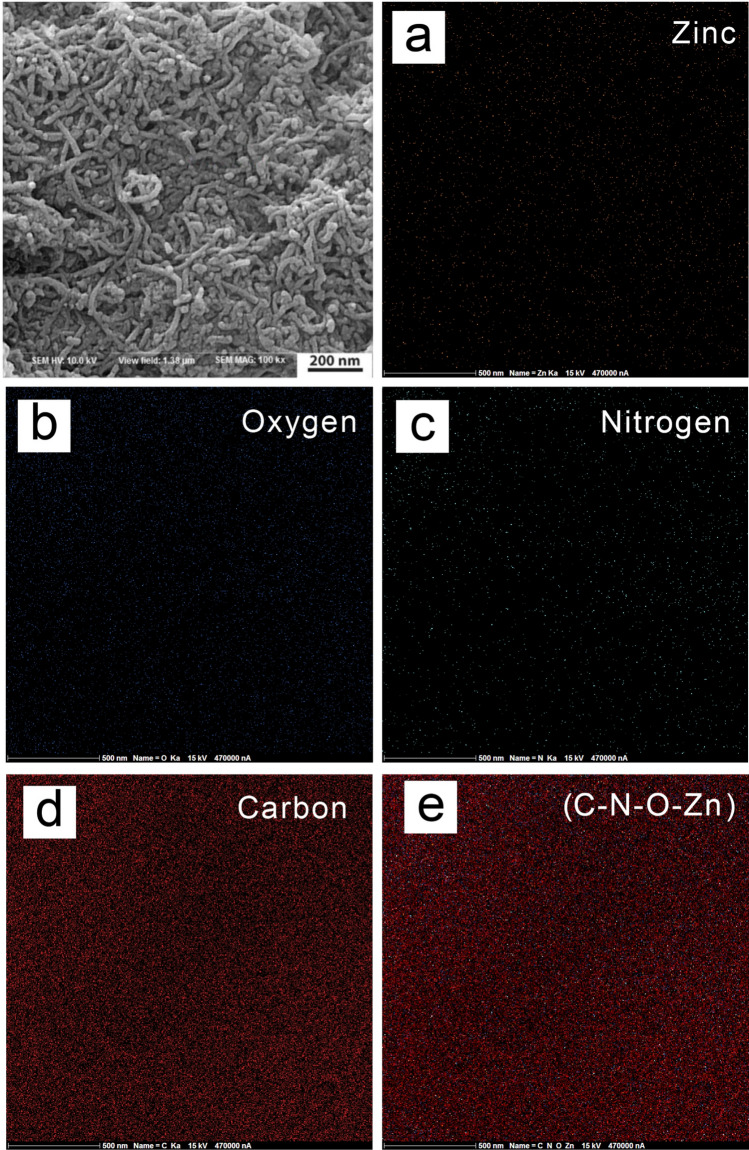
Elemental mapping of MWCNTs/ZnO/Chitosan nanocomposite.

**Figure 4:**
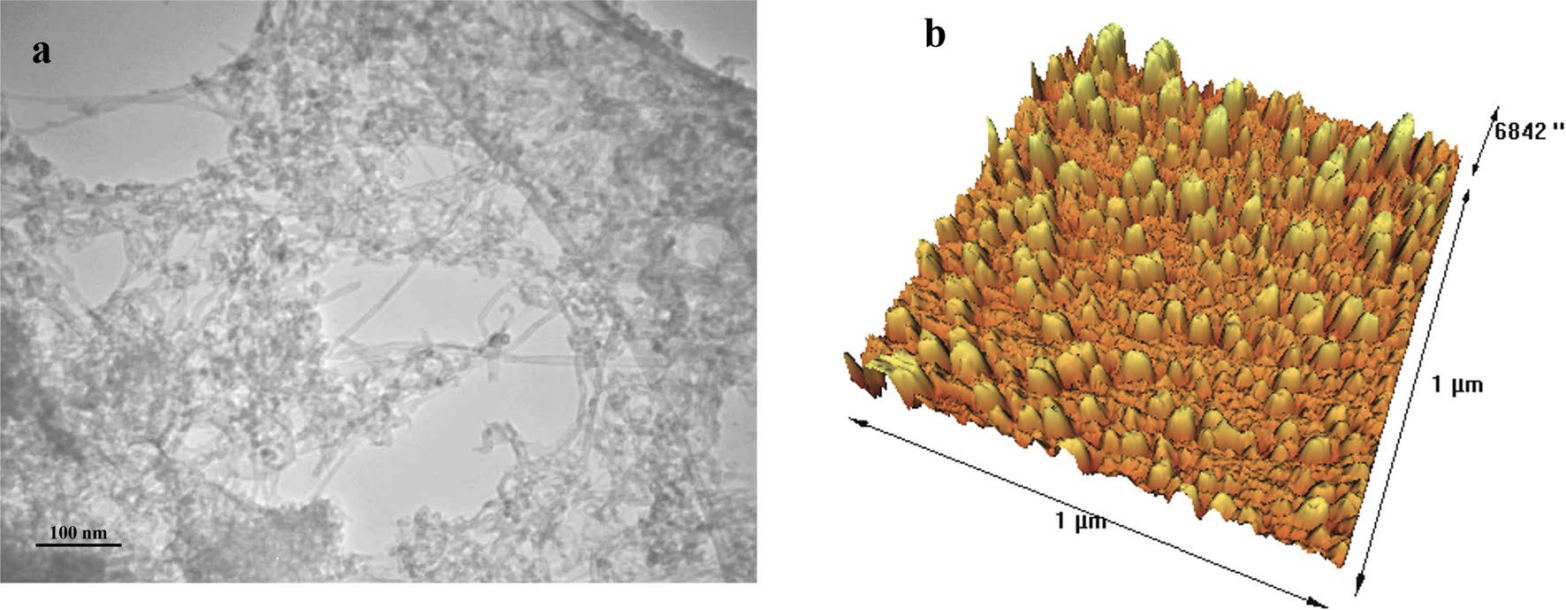
(**a**) TEM images of MWCNTs/ZnO/Chitosan nanocomposite, (**b**) AFM 3D image of MWCNTs/ZnO/Chitosan nanocomposite.

The elemental mapping of MWCNTs/ZnO/Chitosan nanocomposite is depicted in Fig. 3, which indicates the uniform spread of Zn, O, N and C elements. Homogeneous distribution of these elements, which are detectable in the sample, shows the perfect functionalizing of MWCNTs with ZnO with the uniform spread of MWCNTs and Chitosan in this nanocomposite. TEM image of MWCNTs/ZnO/Chitosan represented in Fig. 4a is correspondent with its FESEM image. According to this image, MWCNTs are attached to Chitosan and also, the outer surfaces of MWCNTs are decorated with ZnO nanoparticles perfectly. The deposition of nanoparticles on the surface of MWCNTs may be interpreted for the following reason. Firstly, the acid treatment of original MWCNTs in the mixture solution of HNO_3_ resulted in the functionalization of the outer surface of MWCNTs with oxygen-containing groups like hydroxyl (–OH) and carboxylic acid (–COOH). Also, the FT-IR analysis confirmed the existence of these functional groups. So, conjugation of ZnO nanoparticles to the MWCNTs was due to electrostatic interaction between Zn^2+^ and oxygen involving groups like –OH and –COOH. Atomic force microscopy (AFM) analysis was done by three-dimensional topographic image, to explore the surface properties and morphology of MWCNTs/ZnO/Chitosan nanocomposite. AFM images of MWCNTs/ZnO/Chitosan are depicted in Fig. 4b. The results showed the high surface roughness of produced nanocomposite, which was due to the presence of ZnO nanoparticles on the MWCNTs surface. It was confirmed that MWCNTs spread uniformly on the Chitosan and ZnO nanoparticles have been located on MWCNTs. The results as mentioned above, were consistent with the FESEM and TEM results and illustrated the formation of MWCNTs/ZnO/Chitosan nanocomposite.

#### FT-IR analysis

**Figure 5:**
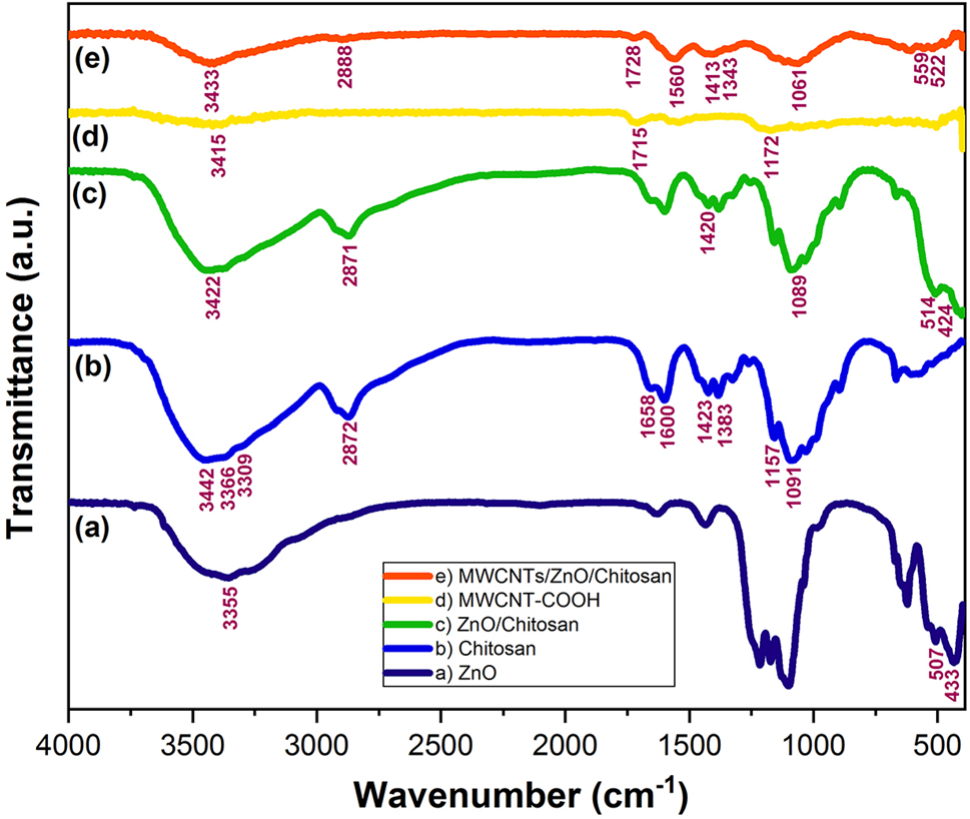
FT-IR spectra of (**a**) pure ZnO, (**b**) pure Chitosan, (**c**) ZnO/Chitosan nanocomposite, (**d**) MWCNT-COOH and (**e**) MWCNTs/ZnO/Chitosan nanocomposite.

To determine the changes in the functional groups during the synthesis of nanocomposites, FT-IR analysis was performed. The FT-IR spectra for ZnO, Chitosan, MWCNT-COOH, ZnO/Chitosan and MWCNTs/ZnO/Chitosan are represented in Fig. 5. The characteristic peaks of ZnO that were observed at 433 and 507 cm^−1^, attributed to the stretching ZnO bond and the broad adsorption peak about 3355 cm^−1^ is related to the surface hydroxyl of ZnO (Fig. 5a). The pure Chitosan spectra in Fig. 5b, exhibited bands at 3250–3450 cm^−1^, which resulted from stretching vibration of –OH and –NH groups. The band at 2872 cm^−1^ showed the asymmetric stretching vibration of –CH group and 1658 cm^−1^ were related to the amide I group. Also, the 1600 cm^−1^ peak was attributed to the –NH deformation. The peaks at 1423 cm^−1^ and 1383 cm^−1^ represented the C–N axial deformation amine group and COOH group of carboxylic acid salt respectively, while 1423 cm^−1^ indicated a small shoulder peak of β (1–4) glycosidic bond in the polysaccharide unit. Moreover, 1089 cm^−1^ was due to the stretching vibration of C–O–C in the glucose ring^36^. Furthermore, the bands at 424 and 514 cm^−1^ were because of the stretching mode of O–Zn–O group^25^. The ZnO/Chitosan FT-IR spectrum exhibited distinguished properties compared to pure Chitosan (Fig. 5c). The main differences were as follows: The broad peak at 3442 cm^−1^ corresponded to the stretching vibration of hydroxyl, amide and amine groups, moved considerably to lower wavenumbers of 3422 cm^−1^ that showed the interaction between this groups and ZnO^37^. This is feasible because of the decreasing of inter and intramolecular hydrogen bonds formed in the interaction with nanoparticles during the formation of nanocomposite^38^. Also, the FT-IR spectrum of Chitosan indicated some characteristics of amide groups I and II, which were present at 1600–1658 cm^−1^. The intensity reduction of these NH_2_ groups bands at 1600–1658 cm^−1^, 3300–3500 cm^−1^ and 700–800 cm^−1^ was the indication of ZnO immobilization upon the Chitosan. The peak situated at 1091 cm^−1^ attributed to C–O stretching, shifted to lower wavenumber 1089 cm^−1^^37^. Furthermore, the peak seen at 1423 cm^−1^ assigned to C–N has shifted to 1420 cm^−1^^38^. Figure 5d,e present the FT-IR spectra of MWCNT-COOH and MWCNTs/ZnO/Chitosan nanocomposite respectively. The weak adsorption band about 1715 and 1172 cm^−1^ for MWCNT-COOH can be assigned to the vibrations of C=O and C–O functional groups, while a powerful and wide absorption band at 3415 cm^−1^ is due to the O–H stretching band of carboxylic acid on the MWCNTs surface^39^. Moreover, the FT-IR spectrum of MWCNTs/ZnO/Chitosan nanocomposite involved characteristic peaks of all the raw substances such as ZnO, Chitosan and MWCNT-COOH^40^. In the spectrum of MWCNTs/ZnO/Chitosan, diverse characteristic bands appeared at 3433, 2888, 1728, 1560, 1413, 1343 and 1061 cm^−1^, which were characteristic bands of Chitosan^41^. For example, the band at 3433 cm^−1^ is because of the stretching of the O–H group of Chitosan, while the band at 2888 cm^−1^ was for C–H stretching and the bands at 1728, 1061 and 1560 cm^−1^ were appeared due to stretching vibrations of C=O and N–H groups in Chitosan. In addition, the characteristic bands at 1413 and 1343 cm^−1^, attributed to stretching vibration of secondary (CH-OH) and primary (CH_2_-OH) alcoholic structure in Chitosan. It is noteworthy, that the identical vibration bands related to pure Chitosan and ZnO were seen in the FT-IR spectrum, which confirmed the existence of Chitosan and ZnO on MWCNTs/ZnO/Chitosan nanocomposite^41^. These findings indicated that ZnO was deposited on MWCNTs through the formation of the zinc carboxylate group. In the lower frequency situation, the bands range from 460 to 559 cm^−1^ in the entire spectra illustrated the presence of the stretching position of the Zn–O group^11^.

#### XRD analysis

**Figure 6:**
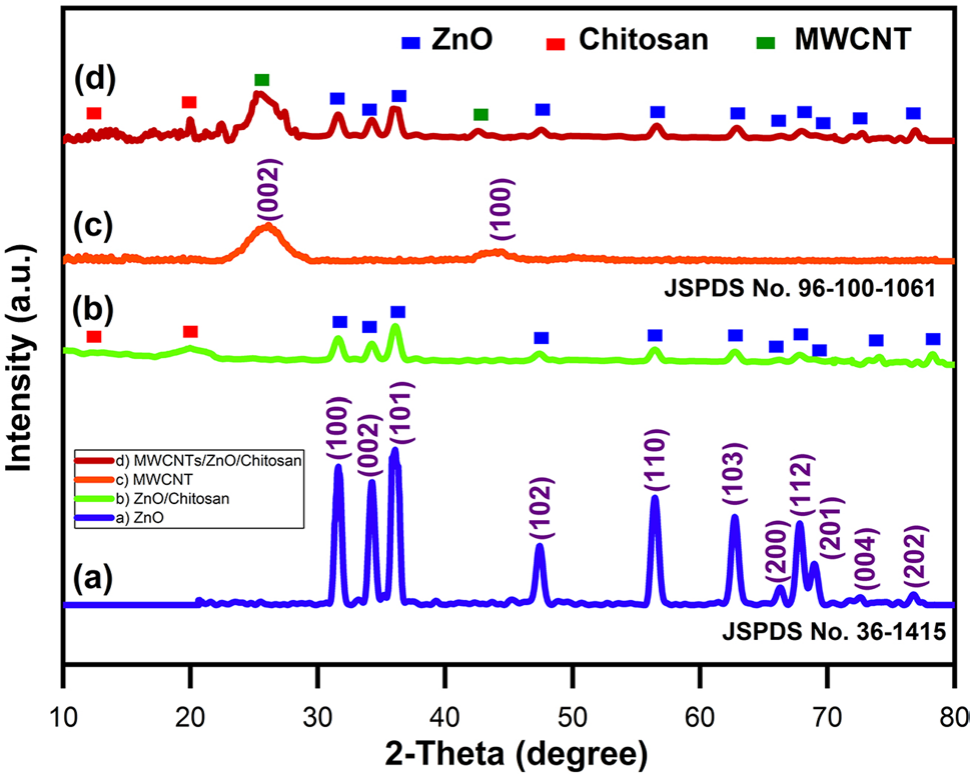
XRD patterns of (**a**) pure ZnO, (**b**) ZnO/Chitosan nanocomposite, (**c**) pure MWCNTs and (**d**) MWCNTs/ZnO/Chitosan nanocomposite.

The XRD analysis has been done to investigate the crystal structure and the phase of all samples. The XRD patterns of the ZnO nanoparticles, MWCNTs, ZnO/Chitosan and MWCNTs/ZnO/Chitosan nanocomposites are indicated in Fig. 6. The obtained XRD spectrum in Fig. 6a showed characterization of pure ZnO NPs by diffraction peaks at 31.62°, 34.27°, 36.07°, 47.42°, 56.47°, 62.72°, 66.32°, 67.82°, 68.97°, 72.57° and 76.77°, related to planes of ZnO crystal at (100), (002), (101), (102), (110), (103), (200), (112), (201), (004) and (202), respectively^42^, which illustrated wurtzite structure spherical phase of ZnO and JCPDS card NO. 36-1415^43^. No additional diverse peaks were observed in obtained spectrum, showing that the ZnO nanostructures were pure. The XRD pattern of ZnO/Chitosan has indicated the major peaks which correspond with both ZnO and Chitosan (Fig. 6b). With regards to previous studies, Chitosan has shown two particular peaks at 2Ɵ = 9.38° and 19.89°, which are typically the fingerprints of semicrystalline Chitosan and are originated from the carbon skeleton of Chitosan. As observed in Fig. 6b, the Chitosan peaks have gotten weaker and shifted to 10.12 and 19.97 may reflect the coordination reaction happening between Zn^2+^ and Chitosan, which, in turn, leads to changing the chitosan crystal. Nevertheless, the respective peaks intensity in XRD pattern of ZnO/Chitosan decreased as compared with pure ZnO and Chitosan. This phenomenon can be attributed to the interactions between Chitosan and ZnO functional groups. It can be concluded that the crystal structure of ZnO was transformed to hexagonal structure after the interaction with Chitosan and observed peaks maintained similar after this structural transition. This observation was also confirmed in SEM results. The emerging of the two sets of diffraction peaks which correspond to ZnO and Chitosan approves the successful formation of ZnO/Chitosan nanocomposite. Figure 6c indicated a sharp diffraction peak at 2Ɵ of 26.13° for the XRD of pristine MWCNTs because of the reflection of C (002) planes of graphite (JSPDS = 96-101-1061). In addition, the MWCNTs indicated another diffraction peak at 2Ɵ of 43.94° which was due to the diffraction of C (100) planes of graphite (JCPDS = 96-100-1061)^41^. The above results approved the high purity of used MWCNTs which were identical to JCPDS card: NO. 96-101-1061^41^. In Fig. 6d, the XRD pattern of MWCNTs/ZnO/Chitosan nanocomposite represented the same diffraction patterns for ZnO nanoparticles, Chitosan and MWCNTs which, in turn, showed that they successfully formed a nanocomposite structure^44^. It was concluded that the ZnO nanoparticles with crystalline structure had been located successfully on the outer surface of functionalized MWCNTs. In comparison with pure ZnO, the position of diffraction peaks at the full width at half maximum of ZnO/Chitosan and MWCNTs/ZnO/Chitosan have been shifted, which illustrate some changes in the ZnO nanoparticles size and shape which formed after nanocomposite formation. The average crystalline size was measured by using the Scherrer formula^27^, and it was found to be 10.47 nm, 12 nm and 30.83 nm for ZnO nanoparticles, ZnO/Chitosan and MWCNTs/ZnO/Chitosan nanocomposites, respectively. The crystal size of samples has been measured and exhibited in Table 1.

**Table 1:** Comparison of particle size of the applied photocatalyst samples.

Sample	2Ө (deg.)	FWHM (rad.)	Diameter (nm)
ZnO	36.05	0.8	10.47
ZnO/Chitosan	36.09	0.7	12
MWCNTs/ZnO/Chitosan	12.8	0.26	30.83

#### BET analysis

**Figure 7:**
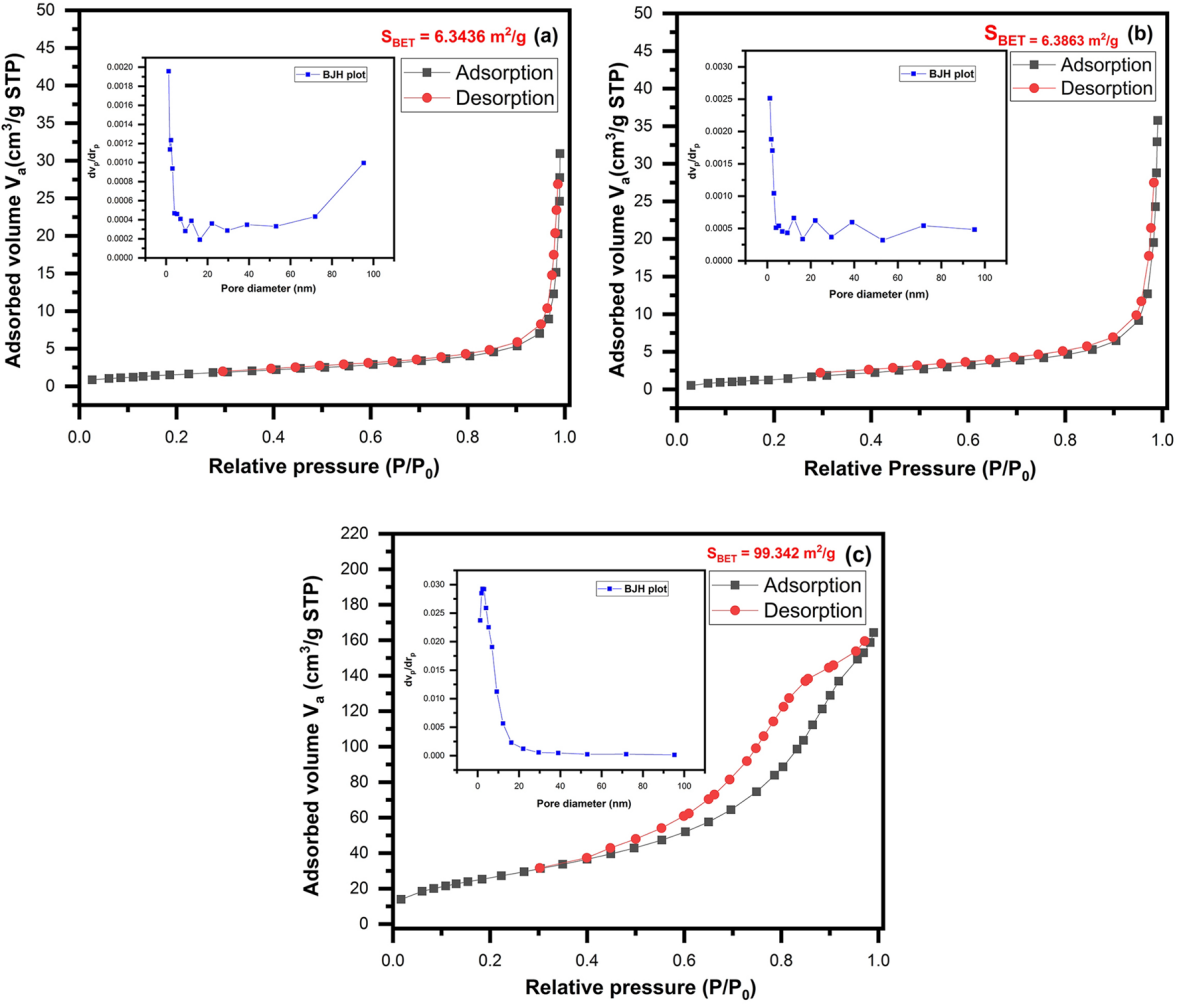
Nitrogen adsorption–desorption isotherms of (**a**) ZnO, (**b**) ZnO/Chitosan and (**c**) MWCNTs/ZnO/Chitosan, Inset: pore size distribution of the prepared samples from the adsorption isotherm measurement.

The Brunauer–Emmett–Teller (BET) analysis indicated the pore diameter and surface area of applied nanostructures. The adsorption capacities of the prepared samples were investigated by N_2_ adsorption–desorption measurements. Nitrogen adsorption–desorption isotherms of the synthesized pure ZnO nanoparticles, ZnO/Chitosan and MWCNTs/ZnO/Chitosan nanocomposites are displayed in Fig. 7a–c and the data of results from the BET equation is shown in Table 2. Based on the IUPAC categorization, all adsorption–desorption isotherms showed a type IV curve with H_3_ hysteresis loops, that confirmed the existence of mesoporous structure having diameters between 2 and 50 nm. This is desirable for smooth ionic diffusion process to occur. According to Table 2, ZnO/Chitosan nanocomposites have enhanced the surface area a little compared to pure ZnO, but MWCNTs/ZnO/Chitosan has a large particular surface area. The BET surface area of ZnO, ZnO/Chitosan and MWCNTs/ZnO/Chitosan were 6.3436, 6.3863 and 99.342 m^2^/g, respectively. The Fig. 7, illustrated the matching pore size distribution plots, which are measured from the adsorption branch of nitrogen isotherms and the Barrett–Jayner–Halenda (BJH) method. The mean pore diameter for ZnO, ZnO/Chitosan and MWCNTs/ZnO/Chitosan were 28.873, 33.415 and 10.206 nm, respectively. The obtained results indicated the highest increase in pore volume of MWCNTs/ZnO/Chitosan ternary nanocomposite. Also, it has shown that after adding MWCNTs, the amount of adsorbed N_2_ significantly increased (up to 0.2535 cm^3^/g) from 0.0 to 0.9 for P/P_0_ and indicated that pore volumes highly increased. So, MWCNTs/ZnO/Chitosan could have much bigger surfaces and the pore volumes than other samples and also showed the crucial reduction in the pore diameter in comparison with ZnO and ZnO/Chitosan. The increase of particular surface area in MWCNTs/ZnO/Chitosan nanocomposites creates the feasibility of increasing the surface-active sites and causes the charge transport. Following, MB molecules are adsorbed by the protonated groups and the electrophilic surface groups of MWCNTs. This structure would accelerate the degradation of dye molecules during the photocatalytic procedure, which lead to superior photocatalytic performance in MWCNTs/ZnO/Chitosan nanocomposite. Both increase in surface area and decrease in pore size resulted to pore volume increase which improved adsorption process by MWCNTs/ZnO/Chitosan.

**Table2:** Surface area, volume and pore size distribution of ZnO, ZnO/Chitosan and MWCNTs/ZnO/Chitosan from nitrogen adsorption–desorption isotherm measurements.

Samples	Surface area BET (S_BET_) (m^2^/g)	Pore size (nm) BJH_ads_	Total pore volume (P/P_0_ = 0.990) (cm^3^/g)
ZnO	6.3436	28.873	0.04579
ZnO/Chitosan	6.3863	33.415	0.05335
MWCNTs/ZnO/Chitosan	99.342	10.206	0.2535

### Optical and electronic characteristics of the ZnO, ZnO/Chitosan and MWCNTs/ZnO/Chitosan nanocomposites

**Figure 8:**
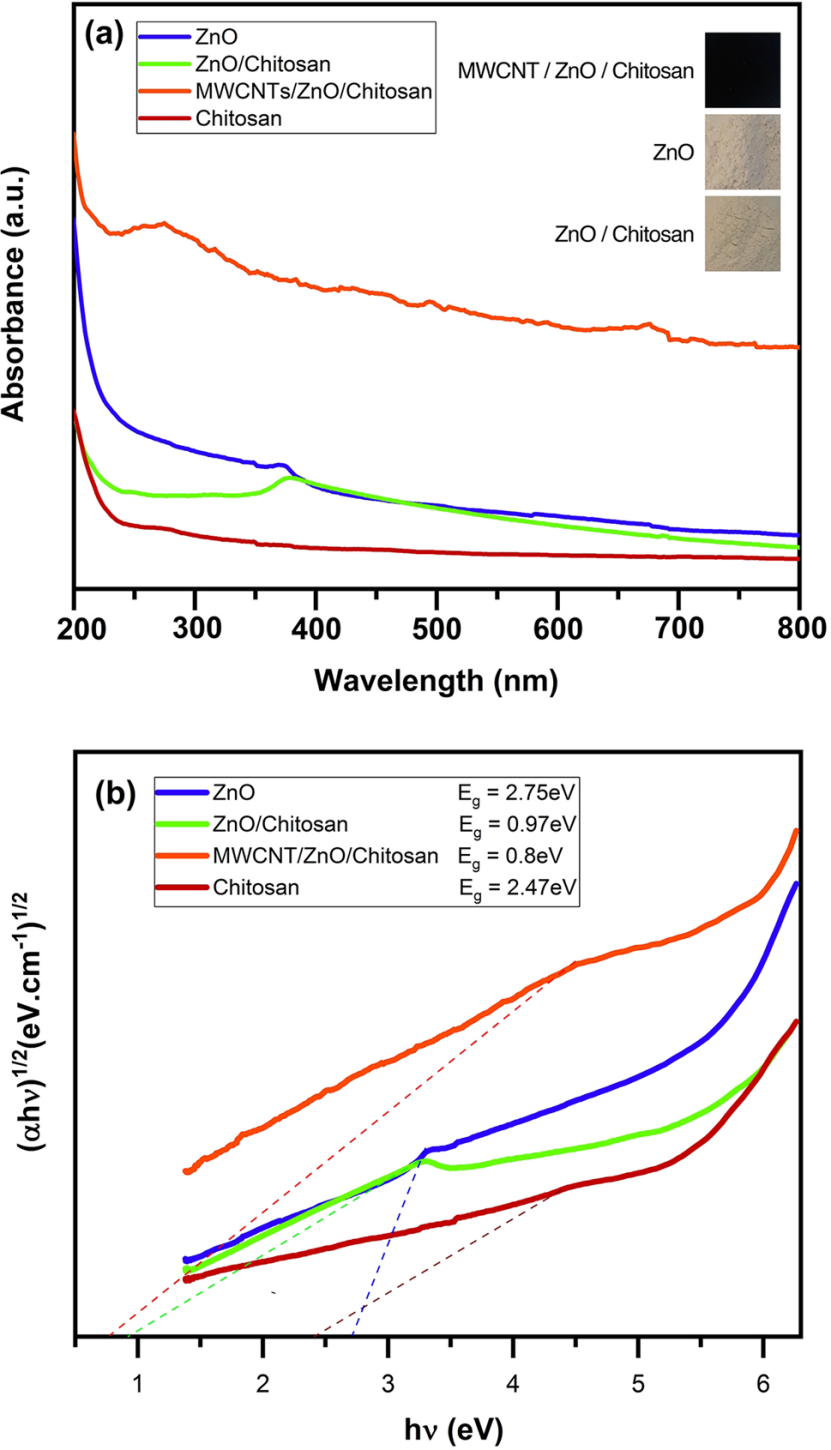
(**a**) UV–Vis diffuse reflecting spectra, (**b**) the bandgap energies of ZnO, Chitosan, ZnO/Chitosan and MWCNTs/ZnO/Chitosan.

The UV–Visible spectra and energy bandgap (E_g_) of synthesized samples are illustrated in Fig. 8a,b. It was observed in Fig. 8a, that the absorbance edge of pure ZnO was situated at 369.2 nm. With comparison of four samples absorbance spectra, it was concluded that MWCNTs/ZnO/Chitosan nanocomposite has a more suitable absorption in the UV area than ZnO nanoparticles and ZnO/Chitosan nanocomposite. Based on the absorption spectra, the bandgap of the samples was measured from the Tauc equation as follows^45^:

2 $$\left( {\upalpha {\text{h}}\upnu \, } \right)^{{{1}/{2}}} = \, \upbeta \, ({\text{h}}\upnu - {\text{ E}}_{{\text{g}}} ).$$

In this equation, β is the indirect bandgap constant, whereas α and hν stand for the optical absorption coefficient and Planck constant^46^. The diagram curve was drawn as the (αhν)^1/2^ vs. hν. The bandgap energy of the synthesized samples and the linear part of obtained plots correspond to the indirect bandgap (E_g_) value when extrapolating to the zero and the obtained results are shown in Fig. 8b. Thus, the indirect band gaps energy of ZnO, Chitosan, ZnO/Chitosan and MWCNTs/ZnO/Chitosan were determined to be 2.75, 2.47, 0.97 and 0.8 eV, respectively. The calculated bandgap values of Chitosan and ZnO nanoparticles were comparable with previous studies^47,48^, respectively. The results obviously showed that after adding Chitosan to ZnO, the bandgap value reduces gradually and subsequently results to prepare condition for the photocatalytic removal of MB dye under the UV light irradiation. Also, the bandgap of MWCNTs/ZnO/Chitosan nanocomposite was the least among the investigated samples which was due to its higher usage effectiveness of UV light in comparison with ZnO and ZnO/Chitosan. Studying of energy plot of MWCNTs/ZnO/Chitosan nanocomposite confirmed that, MWCNTs have an essential role in promoting the efficient electron transfer, and ZnO and Chitosan are semiconductors that assist in transferring induced electrons from their conduction bands to MWCNTs surface. So, developed MWCNTs/ZnO/Chitosan nanocomposite showed superior optical properties which result to the increased photocatalytic function.

### Photocatalytic degradation activity

The evaluation of the photocatalytic activity of the synthesized catalysts was performed through their degradation activity on MB as the organic target under UV light irradiation. Some parameters, like concentration of the dye, catalyst dosage, pH of the dye solution, temperature and time, were optimized using ZnO nanoparticles as a control.

#### Effect of MB concentration

**Figure 9:**
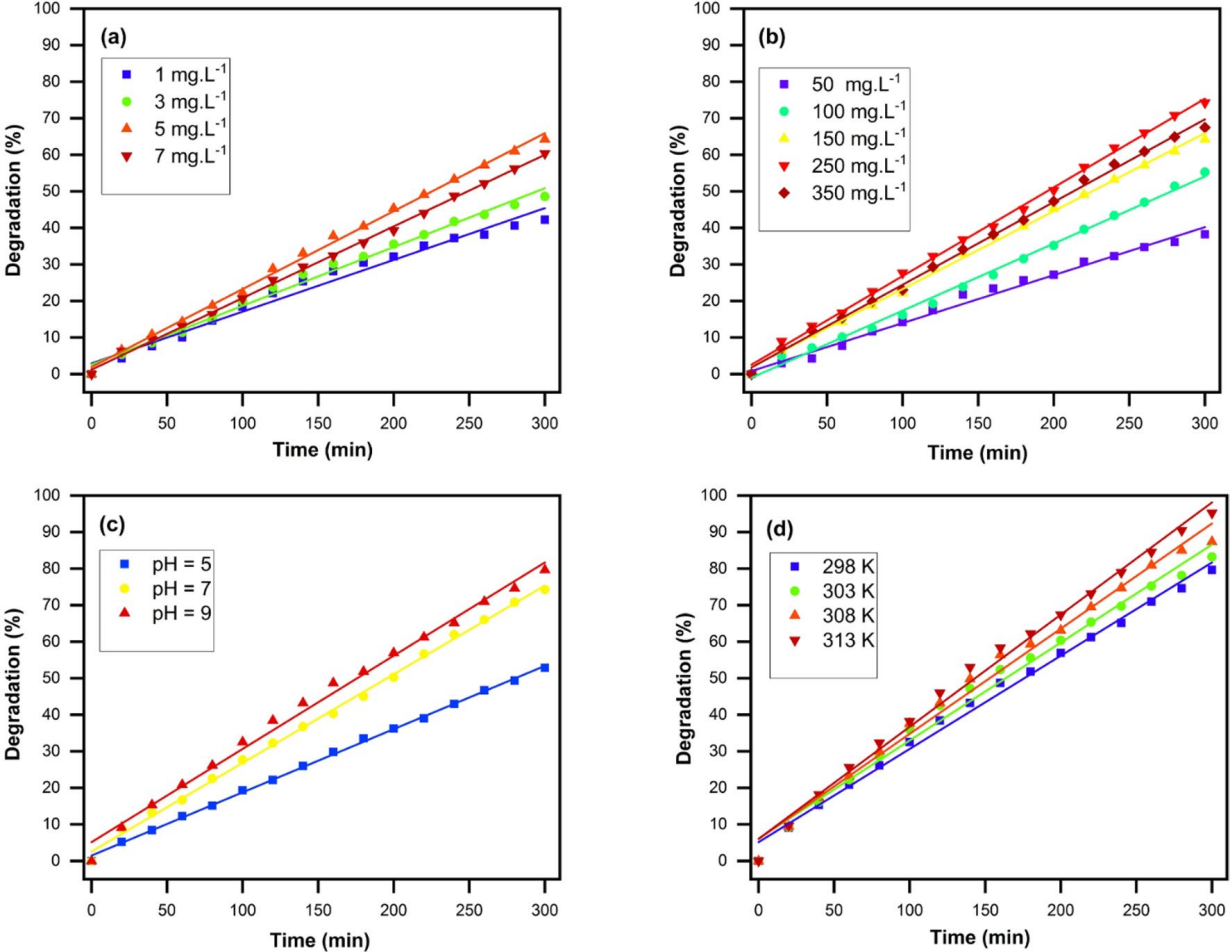
The degradation percentage of MB solution in the presence of ZnO photocatalyst (**a**) with various dye concentrations, (**b**) with different dosage of catalyst, (**c**) with different pH and (**d**) under different temperatures.

To determine the suitable concentration of applied photocatalyst for use in our experiment, investigation has been done to determine its effect on pollutant concentration in a specific range from 1 to7 mg l^−1^ under UV light radiation. The absorbance of all solutions was calculated, and then it was compared with the initial absorption of samples before the photocatalytic process. After removing the percentage, it was designed as a function of MB concentration. Figure 9a represents the obtained results and showed that the maximum obtained photodegradation rate was 64.25% at the concentration of 5 mg l^−1^ of MB. With increasing of MB concentration from 1 to 5 mg l^−1^, we observed that the rate of MB photodegradation was also enhanced. Nevertheless, at the concentration of 7 mg l^−1^, it was observed that the dye degradation was reduced which illustrates, most of the light was screened using the solution and fewer photons were capable of reaching the surface of ZnO nanoparticles. Therefore, the production of electron–hole pairs was highly decreased in this position, because of the oxidizing species absence. As a result, the 5 mg l^−1^ concentration of MB was chosen as the optimal concentration for our further experiment.

#### Effect of photocatalyst concentration

The effect of concentration of photocatalyst on photocatalytic degradation of MB dye solution was evaluated by different nanocomposite catalyst concentrations from 50 to 350 mg l^−1^ in the presence of ZnO photocatalyst. As illustrated in Fig. 9b, the increase of photocatalyst concentration from 50 to 250 mg l^−1^ led to a sharp increment of photodegradation efficiency from 38.23 to 74.25% after 300 min of irradiations, while the efficiency of photodegradation was reduced with more increase of photocatalyst concentration. This enhanced degradation rate with catalyst concentration is because of the increment of available active areas for MB degradation. More increasing of photocatalyst amount may exceed the transmission of light in the suspension system resulting in the reduced photo-irradiation on the surface of the photocatalyst and the apparent accumulation of photocatalysts. This effect led to the efficiency reduction of UV light irradiation. Thus, 250 mg l^−1^ of catalyst was selected as the effective photocatalyst dosage for photocatalytic reaction under the conditions of our experiment.

#### Effect of pH

The pH of the reaction dye solution before and after the photocatalytic reaction were 5.5 and 7, respectively which showed increasing effect of photocatalytic reaction on the pH solution. It has been proposed that adjustment of pH during dye solution is one of the crucial factors^49^. In order to found out the optimum pH, 100 ml MB solution (5 mg l^−1^) was applied and the pH solution was set within three pH values ranging from 5 to 9 by adding suitable amounts of NaOH and HNO_3_ 0.1 M solution. Then, 250 mg l^−1^ of ZnO nanoparticles was added to each solution separately and each container was placed in a magnetic stirrer for 300 min at room temperature. According to the observed results in Fig. 9c, highest dye removal was occurred in pH 9, which originated from the high frequency of OH^−^ in alkaline pH. We assumed that, the surface of the photocatalyst has the negative charge due to the bond between OH^−^ and metal and enhancement of OH^−^ level increase the dye degradation. It should be mentioned that in the acidic pH condition, dye removal showed significant reduction, because, in the acidic pH, the ZnO nanoparticles lose their oxygen through reaction with the hydrogen ion and will be water-soluble (Eq. (3)), and their photocatalyst feature decrease significantly.

3 $${\text{ZnO}}_{(S)}+{2\text{H}}_{(g)}^{+}\to {\text{Zn}}_{(aq)}^{2+}+{\text{H}}_{2}{\text{O}}_{(L)}.$$

#### Effect of the temperature on the MB degradation

The standard dye solution was treated under the best conditions including pH 9, catalyst dosage of 250 mg l^−1^ and 100 ml MB solution at the concentration of 5 mg l^−1^. The above conditions were performed at the temperatures of 298, 303, 308 and 313 K and subjected to UV light for 300 min at the intervals of 20 min and the absorption was recorded with a spectrophotometer. Then, the efficiency of photodegradation was measured and the results presented in Fig. 9d. As observed, the increment of the temperature has enhanced the percentage of degradation. Because of the effective impression of increasing temperature on the amount of MB decolorization of the solution, the degradation reaction can be assumed as an endothermic process. The dye degradation increased by ZnO photocatalyst using increasing temperature which may be due to three major reasons:

Firstly, increasing the temperature causes the molecules to move faster and increases the effective collision between MB and ZnO nanoparticles. Thus, the possibility of degradation of dye molecules on the photocatalyst increases. Secondly, higher temperature result to the high production of free radicals and the formation of bubbles in dye solution. Thirdly, the high temperature decreases the feasibility of electron–hole pair recombination, and hence, the dye degradation of the photocatalyst increases more efficiently.

#### Photocatalytic degradation of MB

**Figure 10:**
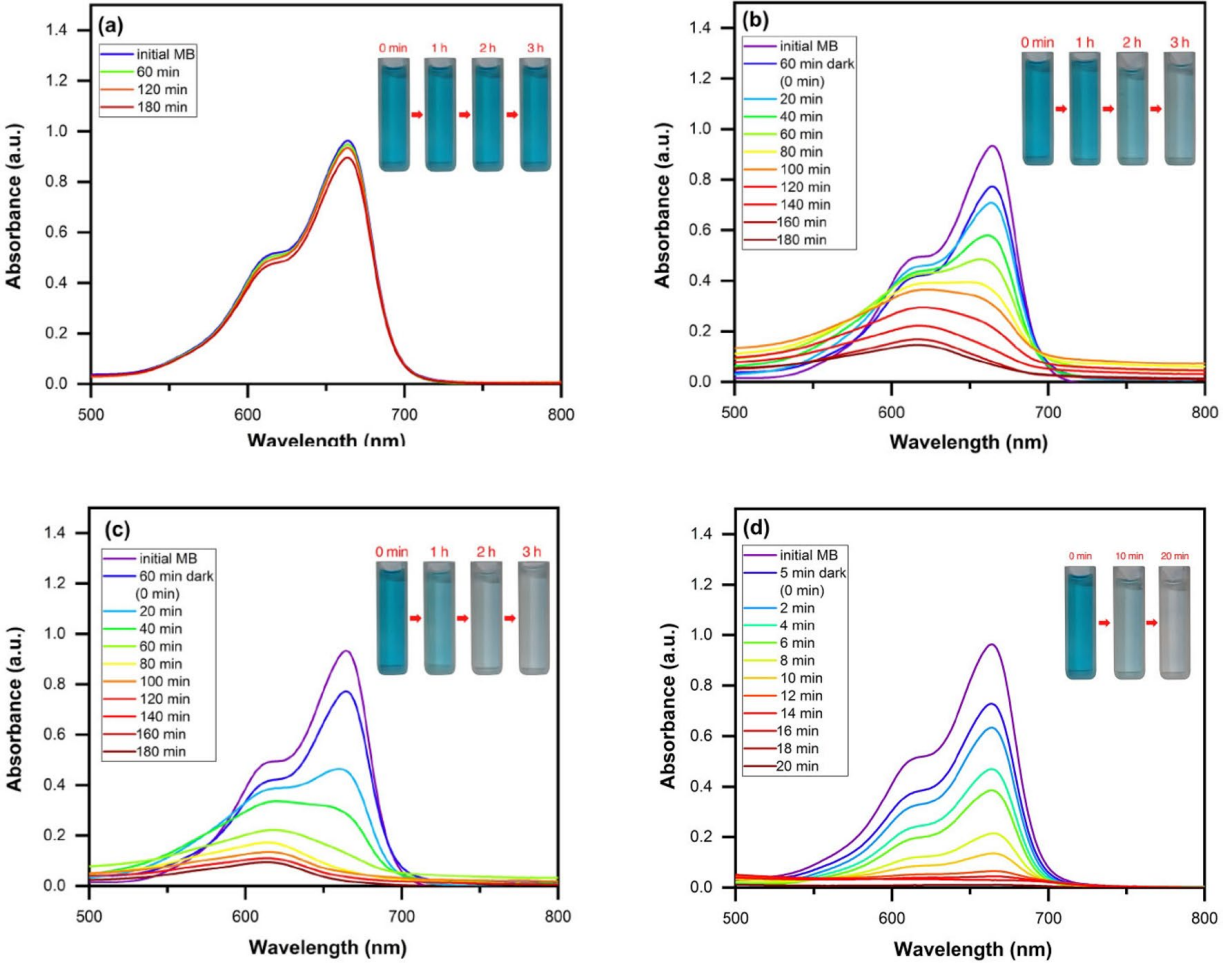
The absorption spectra of UV–Vis for MB dye degradation: (**a**) without catalyst for 180 min, (**b**) ZnO nanoparticles for 180 min, (**c**) ZnO/Chitosan for 180 min and (**d**) MWCNTs/ZnO/Chitosan for 20 min.

The catalytic performance of applied nanostructures was assayed through optimized experiment. Figure 10a–d presents the UV–Vis absorption spectra of MB solution during the photocatalytic process using the ZnO nanoparticles, ZnO/Chitosan and MWCNTs/ZnO/Chitosan nanocomposites under the optimal conditions. ZnO nanoparticles and ZnO/Chitosan nanocomposite were stirred for 60 min in darkness to provide equilibrium of absorption–desorption. Then the photocatalytic properties were measured under UV light every 20 min for the duration of 180 min. However, due to the high photocatalytic properties of the MWCNTs/ZnO/Chitosan nanocomposites, the photocatalytic properties were evaluated for period of 20 min after 5 min in the darkness. The obtained results showed, MWCNTs/ZnO/Chitosan nanocomposites photocatalytic performance was more efficient than the other assayed photocatalysts include ZnO and ZnO/Chitosan, toward MB degradation. Figure 10a indicated the degradation efficiency merely under the UV light irradiation that is investigated without application of any catalysts. The photocatalyst showed that the synthesized MWCNTs/ZnO/Chitosan nanocomposite had more absorption rate of MB compared to other samples, which indicated MWCNTs play an important role in the dye adsorption. Generally, in the dark condition, ~ 17.25% and 17.45% of the initial dye was removed from the aqueous solution containing ZnO and ZnO/Chitosan in 60 min. Also, 9.23% of the initial dye was removed from MWCNTs/ZnO/Chitosan solution in 5 min. The dark adsorption may provide an efficient pretreatment for MB decolorization. Moreover, we also compared the photocatalytic degradation curves of ZnO nanoparticles, ZnO/Chitosan and MWCNTs/ZnO/Chitosan nanocomposites. As shown in Fig. 10b, it was obvious that ZnO nanoparticles could degrade 91.06% of MB dye molecules under UV light irradiation for 180 min. It should be noted that the efficiency of photocatalytic degradation of MB by ZnO is nearly low under UV light irradiation, that is because of its wide bandgap with little usage efficiency of UV light. ZnO/Chitosan showed more suitable photocatalytic function for MB degradation compared with ZnO. Figure 10c represents the ZnO/Chitosan nanocomposite degradation rate which was about 97.67% under irradiation of UV light for 180 min. These results showed that the photocatalytic performance was enhanced after the addition of ZnO to Chitosan. The increased photocatalytic function of ZnO/Chitosan was related to the formation of a type II heterojunction between ZnO and Chitosan interfaces, which is useful to improve the disintegration of prolonging electron-transfer lifespan and photogenerated charges between the interfaces. Finally, it was observed that MWCNTs/ZnO/Chitosan nanocomposites have much higher photocatalytic degradation effect on MB compared with ZnO/Chitosan nanocomposite (Fig. 10d). The results revealed 98.76% degradation of MB dye after exposure to UV light irradiation for 20 min which confirmed the accumulative effect of MWCNTs content on the photocatalytic activity. The obtained results were comparable to the other applied photocatalysts as presented in Table 3. We concluded that our used photocatalyst ternary nanocomposite showed convenient performance in catalytic time and degradation percentage. The results exhibited that suitable bandgap and interfacial engineering of MWCNTs/ZnO/Chitosan had efficient role in increasing the photocatalytic reaction, which incites the highly-efficient separation of photo-generated holes and photo-induced interfacial charge transfer. Also, it was found that interconnections among MWCNTs, ZnO and Chitosan permit a more facile gradual channel electrons transfer.

**Table 3:** Comparison of photocatalytic efficiency of CNT composites with other reported photocatalysts for organic dye degradation.

Catalyst	Light source	Target compound	Photocatalytic degradation%	Time (min)	Ref.
ZnO/CNT	Sun light	Rhodamine B	97	120	^8^
ZnO/CNT	Sun light	Methylene Blue	98	240	^11^
Mn-doped ZnO/CNT	Visible light	Malachite Green	95	120	^44^
N-doped ZnO/CNT	UV light	Methylene Blue	100	60	^7^
ZnO/CNT	Visible light	Malachite Green	79	60	^10^
MWCNTs/ZnO/Chitosan	UV light	Methylene Blue	98.76	20	This work

The perfect photocatalytic function of MWCNTs/ZnO/Chitosan nanocomposite is due to the higher UV wavelength absorption and increased effectiveness of photo-induced electron–hole pairs, which confirmed by obtained UV–Vis spectra and photo electrochemical calculation (Fig. 8). Based on the obtained results, the function of photocatalytic activity may be influenced by the content of MWCNTs, because MWCNTs are efficient electron conductive substances for free radical formation and degradation of MB dye. The efficient performance of photocatalytic would also come from the following reason that the MWCNTs skeleton can alleviate the electron transfer and bandgap pattern of MWCNTs/ZnO/Chitosan nanocomposites which are useful for suppressing the recombination of electron–hole pair and effective electron–hole charge separation. The photocatalytic activity of all applied samples is presented in Table S1.

#### Adsorption assay of MB

**Figure 11:**
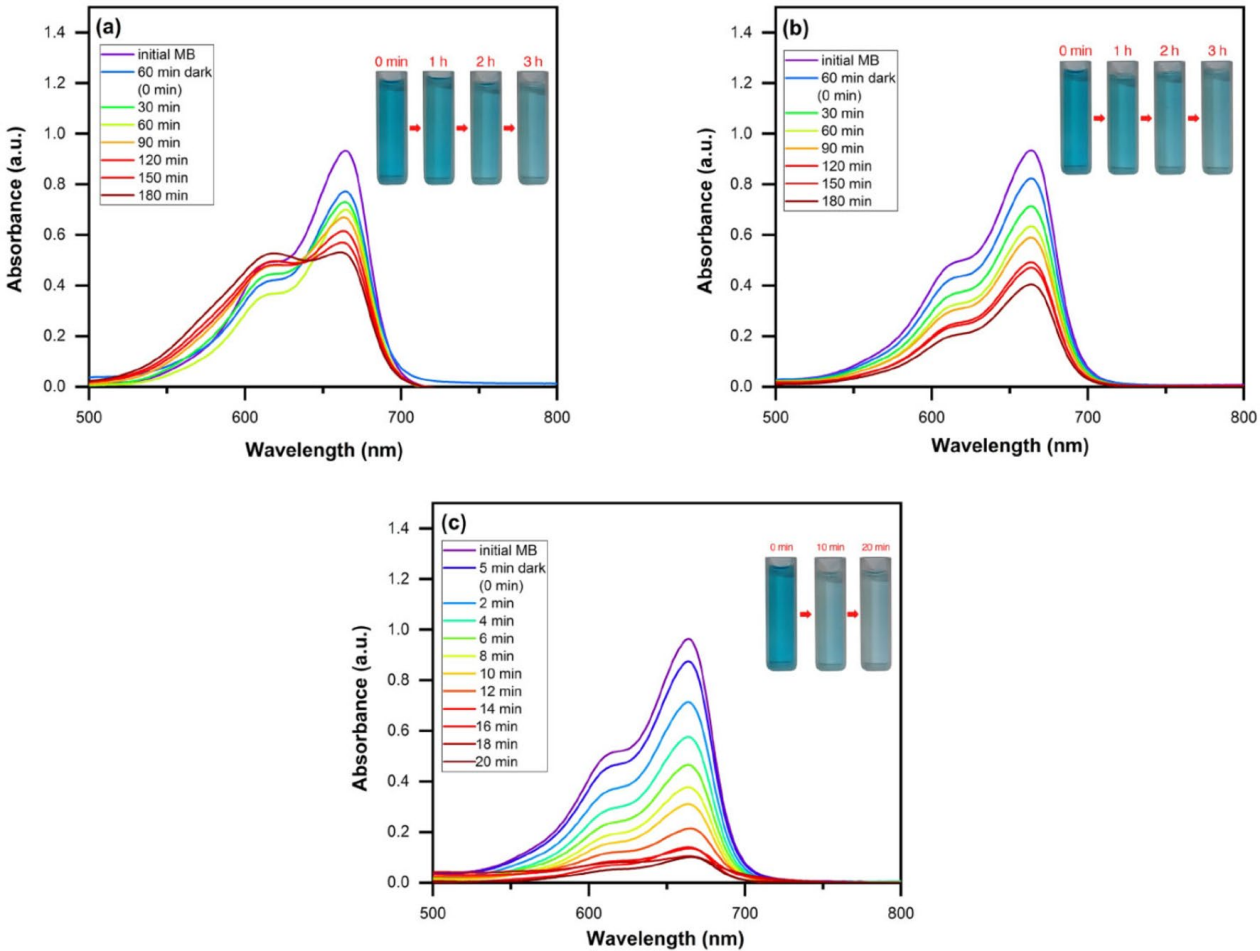
The absorption spectra of UV–Vis for MB dye adsorption by (**a**) ZnO nanoparticles, (**b**) ZnO/Chitosan and (**c**) MWCNTs/ZnO/Chitosan.

**Figure 12:**
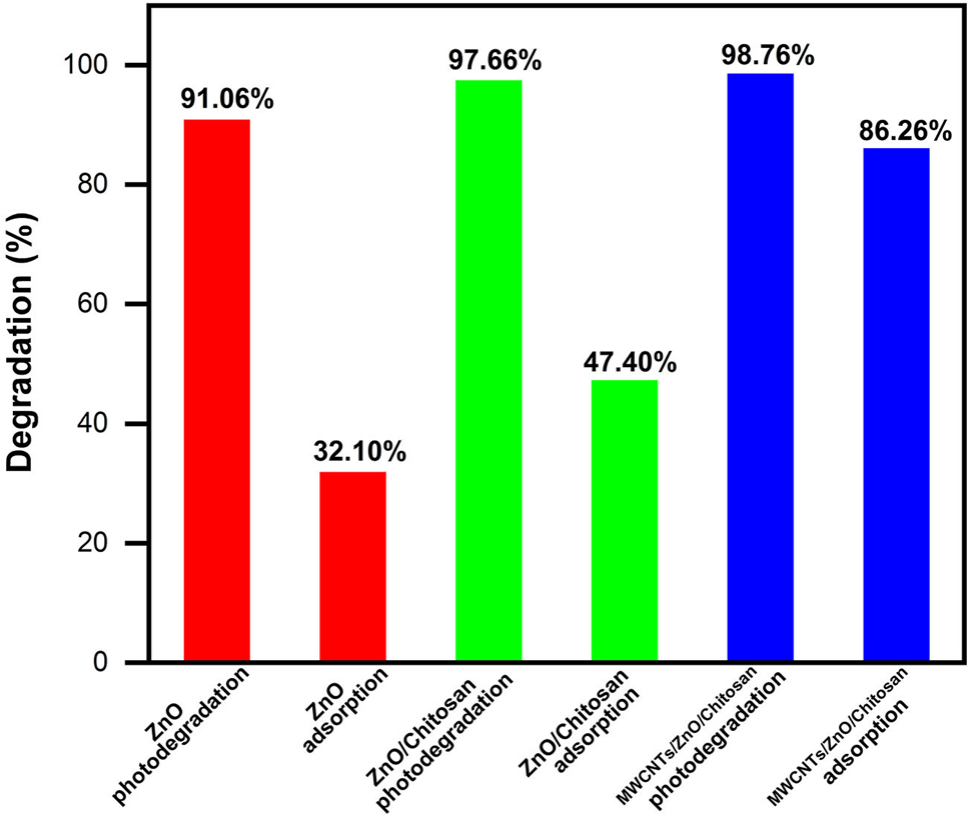
Comparison of MB dye degradation in UV–Vis and adsorption by ZnO, ZnO/Chitosan and MWCNTs/ZnO/Chitosan.

To determine the adsorption rate of applied nanostructures the MB adsorption was determined by the ZnO nanoparticles, ZnO/Chitosan and MWCNTs/ZnO/Chitosan nanocomposites under the optimal conditions (Fig. 11a–c). For investigation of required contact time, the adsorption of MB was done in darkness to prevent any degradation process and to calculate the pure adsorption. The time intervals for the test were selected based on the various changes in MB adsorption. Therefore, we compared the adsorption curves of the applied samples. Clearly, the ZnO nanoparticles and ZnO/Chitosan nanocomposites showed 32.10% and 47.40% of MB dye molecules adsorption during 180 min, respectively. The process of adsorption of ZnO/Chitosan is considerably improved after using MWCNTs. MWCNTs/ZnO/Chitosan could absorb ~ 86.26% of MB dye after 20 min. So, the MB adsorption rates were MWCNTs/ZnO/Chitosan > ZnO/Chitosan > ZnO. The results of the samples were in accordance with BET analysis obtained results in Table 2. As shown, the adsorption properties improved with surface area increase and pore size reduction. Therefore, it can be concluded that with decreasing pore sizes, the number of pores increased and the total pore volume enhanced that creates more surface area for MB adsorption. The adsorption activity of the samples is indicated in Table S2. The data revealed the decrease trend in MB adsorption by increasing contact time because the cavities were filled with MB. Comparison between MB degradation and adsorption of MB using ZnO, ZnO/Chitosan and MWCNTs/ZnO/Chitosan is illustrated in Fig. 12. In the performed comparison, the superiority of the photocatalytic degradation in MWCNTs/ZnO/Chitosan was observed, which could almost degrade 98.76% of the MB pollutants in an aqueous solution.

#### Kinetic of applied reaction

**Figure 13:**
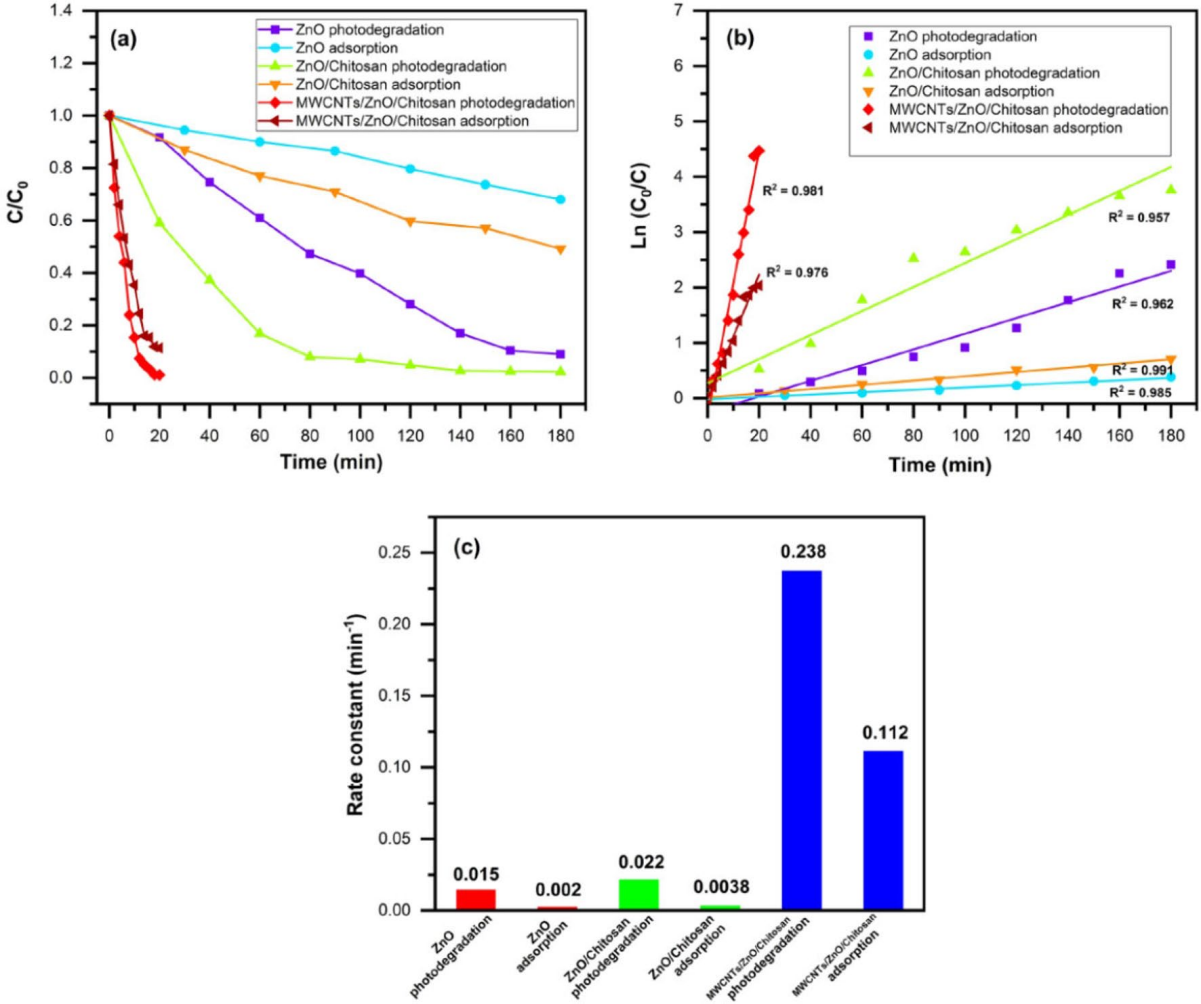
(**a**) photocatalytic degradation and adsorption effectiveness of MB by synthesized samples under UV light and dark conditions, (**b**) the matching kinetic analysis of MB degradation and MB adsorption, (**c**) the kinetic constant of MB degradation as well as MB adsorption by synthesized samples under UV light and dark conditions.

The optimum concentration (C/C_0_) of the MB dye was used with ZnO nanoparticles, ZnO/Chitosan and MWCNTs/ZnO/Chitosan nanocomposites under darkness and UV light irradiation conditions to determine the kinetic of applied adsorption and degradation reactions respectively. It can be observed in Fig. 13a, that MWCNTs/ZnO/Chitosan nanocomposite at the presence of UV light irradiation exhibited the highest photocatalytic reaction compared to other samples. The degradation effectiveness of MB was in present order: ZnO < ZnO/Chitosan < MWCNTs/ZnO/Chitosan and the adsorption efficiencies of MB in darkness condition was also in proper order as follows: ZnO < ZnO/Chitosan < MWCNTs/ZnO/Chitosan. In order to research the kinetics of adsorption and photodegradation of MB the pseudo-first-order of kinetic models were applied, and can be presented by the equation such as ln (C_0_/C) = kt, in which t represents time and k stands for adsorption and photodegradation rate constant respectively, and it can be measured in adjustment with the plots of ln (C_0_/C) vs. time. The rate constant values are shown in Fig. 13b,c for synthesized catalysts under darkness and UV light irradiation conditions. The MWCNTs/ZnO/Chitosan nanocomposite photodegradation possesses the most remarkable rate constant value of 0.238 min^−1^ compared to MWCNTs/ZnO/Chitosan nanocomposite adsorption of 0.112 min^−1^, ZnO/Chitosan nanocomposite photodegradation of 0.022 min^−1^, ZnO/Chitosan adsorption of 0.0038 min^−1^, ZnO photodegradation of 0.015 min^−1^ and ZnO adsorption of 0.002 min^−1^. Thus, the photocatalytic function of MWCNTs/ZnO/Chitosan was much more efficient than ZnO/Chitosan and pure ZnO. Also, the constant rate value of all synthesized samples under UV light irradiation were much more than adsorption in dark conditions, and in addition, the MB dye were decolorized more rapidly and in more quantity under UV light.

### Stability of photocatalyst

**Figure 14:**
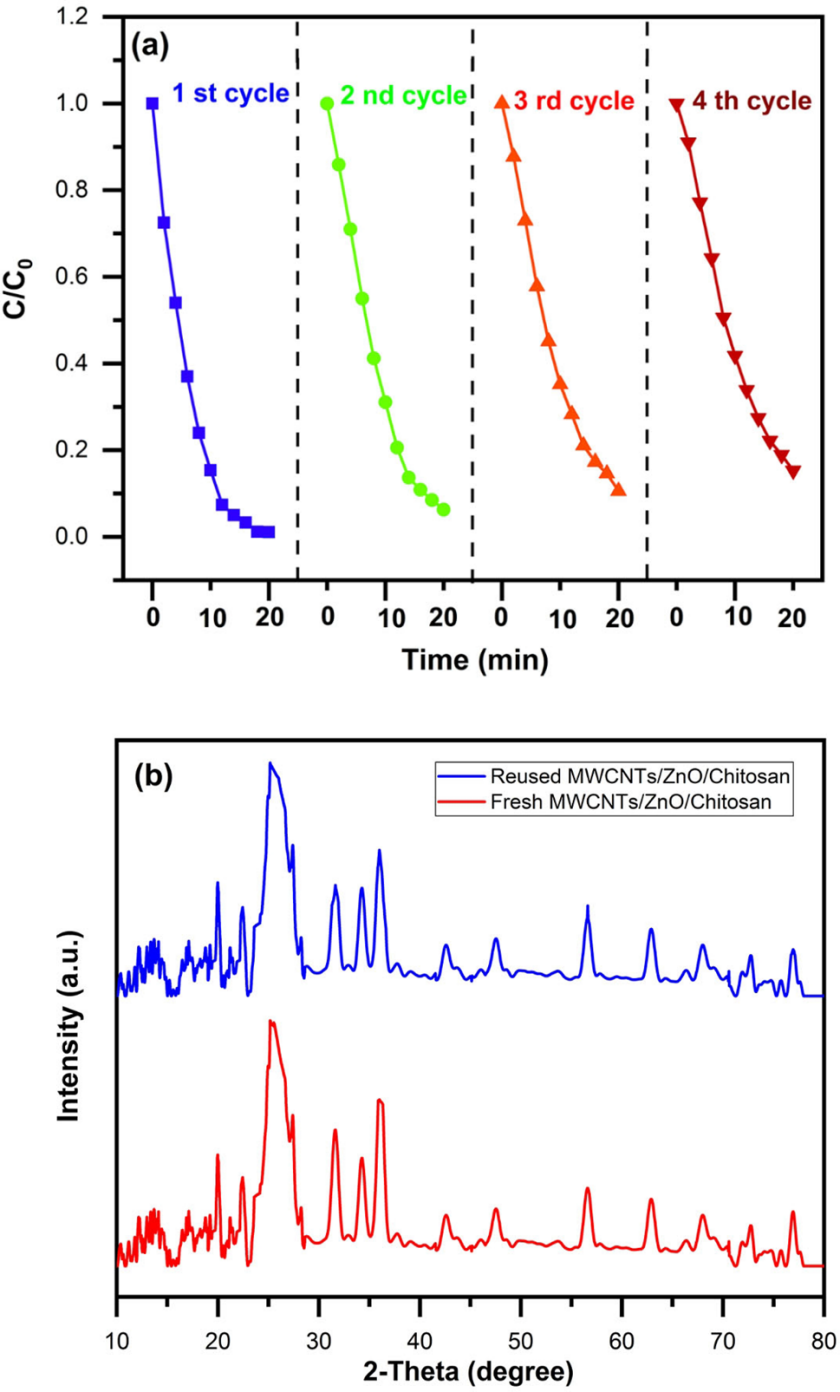
The test of stability of MWCNTs/ZnO/Chitosan: (**a**) recycling experiments of MWCNTs/ZnO/Chitosan for the photocatalytic degradation of MB. (**b**) XRD patterns of MWCNTs/ZnO/Chitosan before and after four consecutive recycling photocatalysis.

Reusability and stability are important parameters for photocatalytic activity since high stability of the catalyst results in lower cost in their industrial applications. We investigated the reusability of photocatalytic degradation function of MWCNTs/ZnO/Chitosan for four successive cycles, which is shown in Fig. 14a. Consequently, in each experiment cycle, we collected the photocatalyst/dye molecules and after washing with deionized water, reapplied them in the next cycle under the same experimental conditions. As a result, the decolorization rate of MB dye declined from 98.76% at the beginning to 85% at the fourth cycle after 20 min. The obtained results obviously showed that the MWCNTs/ZnO/Chitosan nanocomposite present good stability and reusability for practical use in refining the environmental pollutants. Moreover, characterization of MWCNTs/ZnO/Chitosan before and after cycling tests was determined by XRD analysis. It was confirmed that the XRD patterns of MWCNTs/ZnO/Chitosan before and after recycling tests are nearly unchanged (Fig. 14b), which showed that MWCNTs/ZnO/Chitosan had an excellent stability for the photocatalytic degradation of MB. Table 4, represents the turnover number (TON) and turnover frequency (TOF) values for MWCNTs/ZnO/Chitosan ternary nanocomposites in the four continuous cycles in order to depicts the degradation effectiveness of the photocatalyst. The obtained results were in correspondence with the photo-degradation results, and explained that there are still some organic compounds of the previous catalytic experiment that obstruct the pores after each cycle. The MWCNTs/ZnO/Chitosan ternary nanocomposite indicated the highest TOF and TON values. The MB molecules were degraded in 20 min under the irradiation of UV light. According to obtained values of TON and TOF, it was concluded that the photocatalyst was greatly reactive and stable in longer reaction time. Furthermore, it was indicated decrease of the reaction rate in the additional cycles.

**Table 4:** TON, TOF for each reuse using MWCNTs/ZnO/Chitosan.

Parameters	Photocatalytic degradation MB%	Turnover number TON.10^3^	Turnover frequency TOF.10^6^ (S^−1^)
Reuse I	98.85	9.08	7.56
Reuse II	93.61	8.61	7.17
Reuse III	89.31	8.20	6.83
Reuse IV	85	7.81	6.51

*TON* turnover number: mole of degraded MB per mole of active photocatalyst, *TOF* turnover frequency: mole of degraded MB per mole of active photocatalyst after 20 min.

### Photocatalytic mechanism

**Figure 15:**
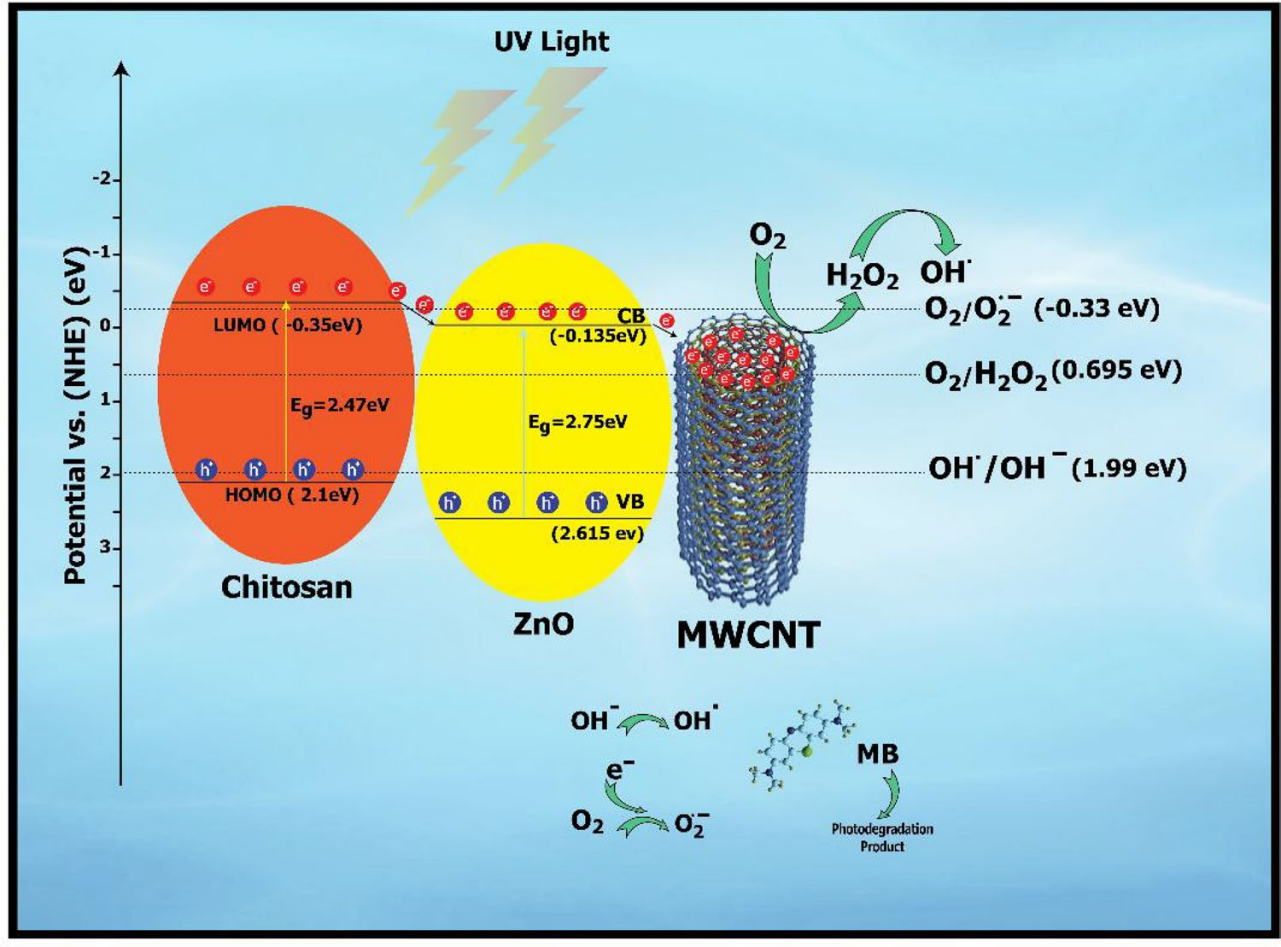
Schematic diagram of proposed photocatalytic mechanism by separation of photo-produced charge carriers in MWCNTs/ZnO/Chitosan ternary nanocomposite.

To find out the increased photocatalytic performance in the presence of MWCNTs/ZnO/Chitosan ternary nanocomposite, the suggested mechanism is illustrated in Fig. 15. The proposed mechanism is based on electron–hole induction and transfer under UV light irradiation. According to results of the UV–Vis diffuse reflecting spectra which were shown in Fig. 8, edge, valence band (VB), conduction band (CB) and the edge potential of Chitosan and ZnO semiconductor were measured by applying the following equations:

4 $${\text{E}}_{{{\text{VB}}}} = {\text{ X }} - {\text{ E}}^{{\text{e}}} + \, 0.{\text{5 E}}_{{\text{g}}} ,$$

5 $${\text{E}}_{{{\text{CB}}}} = {\text{ E}}_{{{\text{VB}}}} {-}{\text{ E}}_{{\text{g}}} .$$

where E_CB_ represent the conduction band edge potential, E_VB_ stand for the valence band edge potential, X is the electronegativity of the semiconductor, E_g_ shows the bandgap of the semiconductor and E^e^ stands for the energy of free electrons on the hydrogen scale which is about 4.5 eV vs. NHE^50^. The electronegativity of ZnO had been reported 5.47 eV^51^. The E_VB_ and E_CB_ of ZnO were measured to be 2.615 eV and − 0.135 eV, respectively. According to Liang et al.,^47^, the LUMO energy level potential (E_LUMO_) of the Chitosan was calculated to be − 0.35 eV. In addition, based on the bandgap of 2.47 eV for the Chitosan that was given before, the E_HOMO_ of the Chitosan was determined 2.1 eV. According to the illustrated scheme in Fig. 15, after exposure of MWCNTs/ZnO/Chitosan nanocomposite under UV light, Chitosan electrons will be excited under UV light because of its less bandgap and produce electron–hole pairs. Therefore, the excited electrons transferred to the LUMO level of Chitosan which leaves holes in the HOMO level. Because the LUMO edge potential of Chitosan (− 0.35 eV) is more negative than conduction band (CB) of ZnO (− 0.135 eV), the electrons in the LUMO level of Chitosan can be transmitted to the CB of ZnO through the built-in heterojunction between Chitosan and ZnO. Because of the heterojunction production, the electron–hole pairs could be separated easily and thus led to the increased photocatalytic function. MWCNTs operate as an electron reservoir and destroy the charge carrier recombination efficiently and increases the photocatalytic function because electrons are quickly transferred to the MWCNTs. By capturing electrons, these transferred electrons in the MWCNTs can reduce O_2_ to H_2_O_2_^52^. Furthermore, a small number of electrons that were moved to the surface of Chitosan could be captured by O_2_ to create ${\text{O}}_{2}^{.-}$. The LUMO level potential of Chitosan, which is − 0.35 eV vs. NHE has more negativity compared with O_2_/${\text{O}}_{2}^{.-}$ (− 0.33 eV vs. NHE). So, the photogenerated electrons that remained on the LUMO level of Chitosan have powerful reducibility and thus react much more with O_2_ to produce ${\text{O}}_{2}^{.-}$ that promote degradation of MB. In addition, the valence band (VB) of ZnO (2.615 eV vs, NHE) and the HOMO level of Chitosan (2.1 eV vs. NHE) has more positivity than ${\text{OH}}^{.}$/${\text{OH}}^{-}$ (1.99 eV vs. NHE). Thus, the created holes on the VB of ZnO and the HOMO of Chitosan oxidize ${\text{OH}}^{-}$ to generate ${\text{OH}}^{.}$, that is equal to the results of the trapping test^53,54^. Therefore, ${OH}^{.}$ radicals produced by electrons that act as an efficient oxidant that react with MB dye molecules and decompose the dye to carbon dioxide and water. Thus, based on the above photocatalytic degradation research, the suggested feasible mechanism is summarized as such:

6 $${\text{MWCNTs}}/{\text{ZnO}}/{\text{Chitosan }} + {\text{ h}}\upnu \, \to {\text{ MWCNTs}}/{\text{ZnO}}/{\text{Chitosan}}\left( {e_{CB}^{ - } + h_{VB}^{ + } } \right),$$

7 $${e}^{-}+{\text{O}}_{2}\to {\text{O}}_{2}^{.-},$$

8 $${\text{O}}_{2}^{.-}+{\text{H}}_{2}\text{O}\to {\text{OH}}_{2}^{-}+{\text{OH}}^{-},$$

9 $${h}^{+}+{\text{OH}}^{-}\to \text{OH},$$

10 $${\text{OH}}^{.}+{\text{O}}_{2}^{.-}+\text{MB}\to {\text{CO}}_{2}+{\text{H}}_{2}\text{O}+\text{oxidation products}.$$

Consequently, a gradual transfer of electrons in MWCNTs/ZnO/Chitosan ternary nanocomposite result to transfer of electron charge via their interfaces efficiently, so increase the photocatalytic effectiveness.

### Antibacterial activity

**Figure 16:**
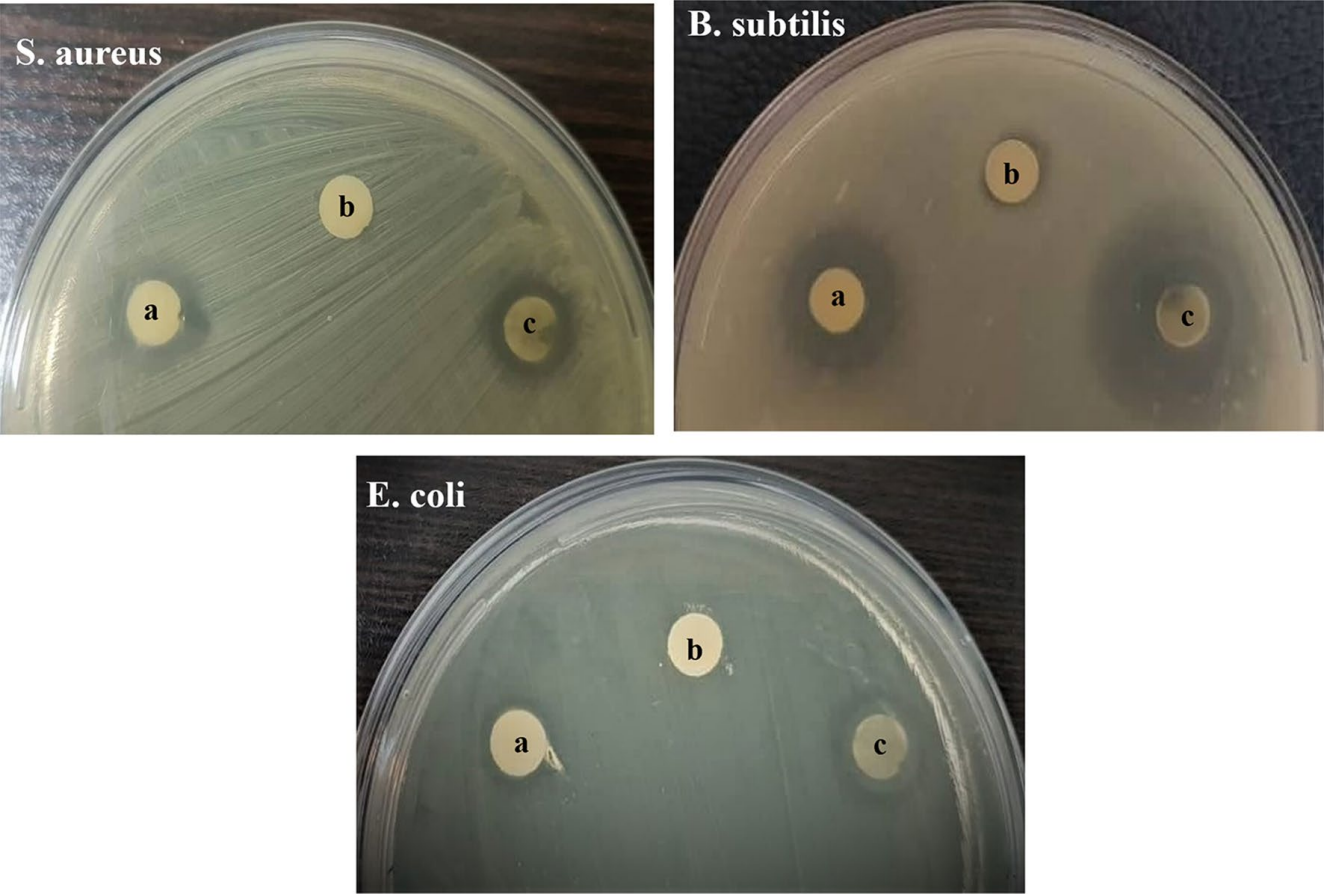
Antibacterial assay of synthesized ZnO (**a**), ZnO/Chitosan (**b**) and MWCNTs/ZnO/Chitosan (**c**) sample discs on *S. aureus*, *B. subtilis* and *E. coli* plates.

The antibacterial property of synthesized nanostructures was assayed through disc diffusion method against three pathogenic gram-positive and gram-negative bacteria. As illustrated in Fig. 16a–c showed the inhibition zones for applied ZnO (a), ZnO/Chitosan (b) and MWCNTs/ZnO/Chitosan (c) which formed on *B. subtilis*, *E. coli* and *S. aureus* strain plates. The diameter of formed clear zones by ZnO, ZnO/Chitosan and MWCNTs/ZnO/Chitosan were 23, 0 and 35 mm for *S. aureus*, 32, 10 and 65 mm for *B. subtilis* and 15, 0 and 31 mm for *E. coli* inoculated media. The ZnO NPs showed efficient antibacterial properties which their effect was inhibited after addition of chitosan layer significantly. The obtained results demonstrated that the highest antibacterial activity was related to MWCNTs/ZnO/Chitosan which was observed in all bacteria. Among applied bacteria, most inhibition effect was induced by *B. subtilis* which is gram positive bacterium.

## Conclusion

In the present study, a MWCNTs/ZnO/Chitosan ternary nanocomposite has been synthesized successfully via a sonication-based strategy. The prepared nanocomposite exhibited higher photocatalytic activity at 98.76% after 20 min for the degradation of MB compared to the ZnO and ZnO/Chitosan nanostructures. The investigation of the proposed mechanism showed that some free radicals (${\text{O}}_{2}^{.-}$ and ${\text{OH}}^{.}$) would be released by MWCNTs/ZnO/Chitosan after excitation by UV light irradiation. Also, the both photocatalytic and antibacterial effects of applied ZnO NPs had been demonstrated previously^55,56^. Similar to the structure of organic dyes, the main ingredients of the bacterial cell wall are organic molecules and are prone to decomposition via redox reactions and their photo-generated reactive specimens. The proposed nanostructures showed efficient application to limit the growth of both pathogenic gram-positive and gram-negative bacteria. Finally, we concluded that our ternary nanocomposite possesses a fantastic ability to reduce the hazardous dyes and kill the pathogenic bacteria existing in the industrial effluents.

## Supplementary Information

Supplementary Information.
