# Supplementary material for: Facile fabrication of ternary MWCNTs/ZnO/Chitosan nanocomposite for enhanced photocatalytic degradation of methylene blue and antibacterial activity
Source: Sci Rep. 2022 Apr 8;12:5927. doi: 10.1038/s41598-022-09571-5 (PMC8993914; doi:10.1038/s41598-022-09571-5)
Supplement: Supplementary file 1 — Supplementary Information. [file 41598_2022_9571_MOESM1_ESM.docx]

**Supplementary data**

| Catalyst | Condition | Catalyst dosage | pH | Time | Absorbance | yield |
| --- | --- | --- | --- | --- | --- | --- |
| ZnO | UV light | 250 mg/L | 9 | 0 min | 0.772 | - |
| ZnO | UV light | 250 mg/L | 9 | 20 min | 0.708 | 8.29% |
| ZnO | UV light | 250 mg/L | 9 | 40 min | 0.576 | 25.38% |
| ZnO | UV light | 250 mg/L | 9 | 60 min | 0.471 | 38.99% |
| ZnO | UV light | 250 mg/L | 9 | 80 min | 0.365 | 52.77% |
| ZnO | UV light | 250 mg/L | 9 | 100 min | 0.308 | 60.10% |
| ZnO | UV light | 250 mg/L | 9 | 120 min | 0.217 | 71.89% |
| ZnO | UV light | 250 mg/L | 9 | 140 min | 0.131 | 83.03% |
| ZnO | UV light | 250 mg/L | 9 | 160 min | 0.081 | 89.50% |
| ZnO | UV light | 250 mg/L | 9 | 180 min | 0.069 | 91.06% |
| ZnO/Chitosan | UV light | 250 mg/L | 9 | 0 min | 0.774 | - |
| ZnO/Chitosan | UV light | 250 mg/L | 9 | 20 min | 0.458 | 40.82% |
| ZnO/Chitosan | UV light | 250 mg/L | 9 | 40 min | 0.289 | 62.66% |
| ZnO/Chitosan | UV light | 250 mg/L | 9 | 60 min | 0.131 | 83.07% |
| ZnO/Chitosan | UV light | 250 mg/L | 9 | 80 min | 0.062 | 91.98% |
| ZnO/Chitosan | UV light | 250 mg/L | 9 | 100 min | 0.055 | 92.89% |
| ZnO/Chitosan | UV light | 250 mg/L | 9 | 120 min | 0.037 | 95.21% |
| ZnO/Chitosan | UV light | 250 mg/L | 9 | 140 min | 0.021 | 97.28% |
| ZnO/Chitosan | UV light | 250 mg/L | 9 | 160 min | 0.020 | 97.41% |
| ZnO/Chitosan | UV light | 250 mg/L | 9 | 180 min | 0.018 | 97.67% |
| MWCNTs/ZnO/Chitosan | UV light | 250 mg/L | 9 | 0min | 0.874 | - |
| MWCNTs/ZnO/Chitosan | UV light | 250 mg/L | 9 | 2min | 0.634 | 27.45% |
| MWCNTs/ZnO/Chitosan | UV light | 250 mg/L | 9 | 4min | 0.475 | 34.75% |
| MWCNTs/ZnO/Chitosan | UV light | 250 mg/L | 9 | 6min | 0.386 | 46.98% |
| MWCNTs/ZnO/Chitosan | UV light | 250 mg/L | 9 | 8min | 0.214 | 70.60% |
| MWCNTs/ZnO/Chitosan | UV light | 250 mg/L | 9 | 10min | 0.135 | 81.46% |
| MWCNTs/ZnO/Chitosan | UV light | 250 mg/L | 9 | 12min | 0.065 | 91.07% |
| MWCNTs/ZnO/Chitosan | UV light | 250 mg/L | 9 | 14min | 0.044 | 93.95% |
| MWCNTs/ZnO/Chitosan | UV light | 250 mg/L | 9 | 16min | 0.029 | 96.01% |
| MWCNTs/ZnO/Chitosan | UV light | 250 mg/L | 9 | 18min | 0.011 | 98.49% |
| MWCNTs/ZnO/Chitosan | UV light | 250 mg/L | 9 | 20min | 0.01 | 98.76% |

**Table S1.** The photocatalytic activity of applied samples under UV irradiation.

| Catalyst | Condition | Catalyst dosage | pH | Time | Absorbance | yield |
| --- | --- | --- | --- | --- | --- | --- |
| ZnO | dark | 250 mg/L | 9 | 0 min | 0.772 | - |
| ZnO | dark | 250 mg/L | 9 | 30min | 0.730 | 5.44% |
| ZnO | dark | 250 mg/L | 9 | 60 min | 0.699 | 9.45% |
| ZnO | dark | 250 mg/L | 9 | 90 min | 0.668 | 13.47% |
| ZnO | dark | 250 mg/L | 9 | 120 min | 0.615 | 20.34% |
| ZnO | dark | 250 mg/L | 9 | 150 min | 0.569 | 26.29% |
| ZnO | dark | 250 mg/L | 9 | 180min | 0.525 | 32.10% |
| ZnO/Chitosan | dark | 250 mg/L | 9 | 0 min | 0.823 | - |
| ZnO/Chitosan | dark | 250 mg/L | 9 | 30 min | 0.712 | 13.36% |
| ZnO/Chitosan | dark | 250 mg/L | 9 | 60 min | 0.633 | 17.79% |
| ZnO/Chitosan | dark | 250 mg/L | 9 | 90 min | 0.589 | 23.50% |
| ZnO/Chitosan | dark | 250 mg/L | 9 | 120 min | 0.492 | 36.10% |
| ZnO/Chitosan | dark | 250 mg/L | 9 | 150 min | 0.470 | 38.96% |
| ZnO/Chitosan | dark | 250 mg/L | 9 | 180 min | 0.405 | 47.40% |
| MWCNTs/ZnO/Chitosan | dark | 250 mg/L | 9 | 0min | 0.874 | - |
| MWCNTs/ZnO/Chitosan | dark | 250 mg/L | 9 | 2min | 0.713 | 18.42% |
| MWCNTs/ZnO/Chitosan | dark | 250 mg/L | 9 | 4min | 0.576 | 20.87% |
| MWCNTs/ZnO/Chitosan | dark | 250 mg/L | 9 | 6min | 0.465 | 36.12% |
| MWCNTs/ZnO/Chitosan | dark | 250 mg/L | 9 | 8min | 0.377 | 48.21% |
| MWCNTs/ZnO/Chitosan | dark | 250 mg/L | 9 | 10min | 0.310 | 57.41% |
| MWCNTs/ZnO/Chitosan | dark | 250 mg/L | 9 | 12min | 0.213 | 70.74% |
| MWCNTs/ZnO/Chitosan | dark | 250 mg/L | 9 | 14min | 0.140 | 80.76% |
| MWCNTs/ZnO/Chitosan | dark | 250 mg/L | 9 | 16min | 0.135 | 81.46% |
| MWCNTs/ZnO/Chitosan | dark | 250 mg/L | 9 | 18min | 0.104 | 85.71% |
| MWCNTs/ZnO/Chitosan | dark | 250 mg/L | 9 | 20min | 0.100 | 86.26% |

**Table S2.** The photocatalytic activity of applied samples under dark condition.


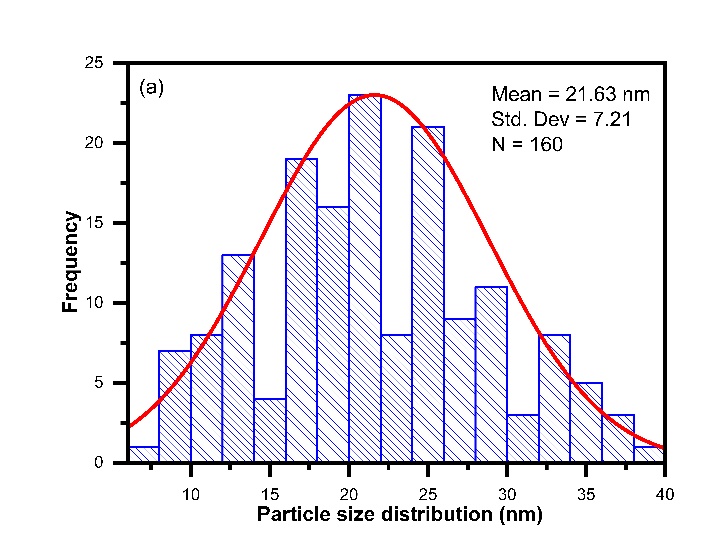

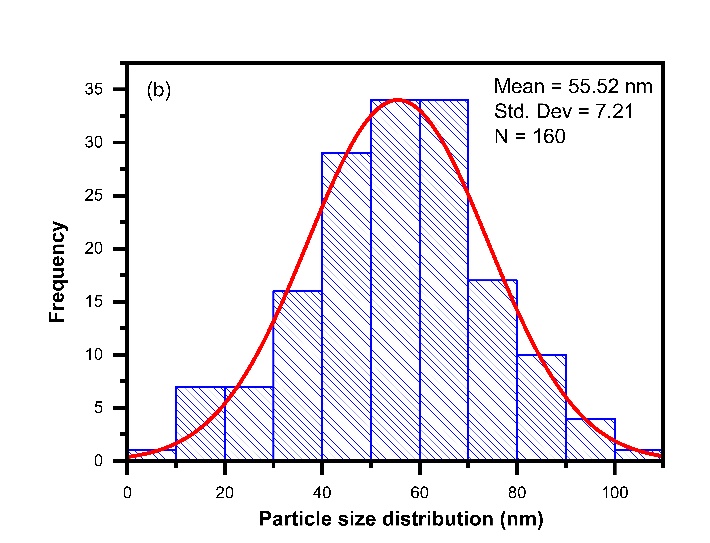


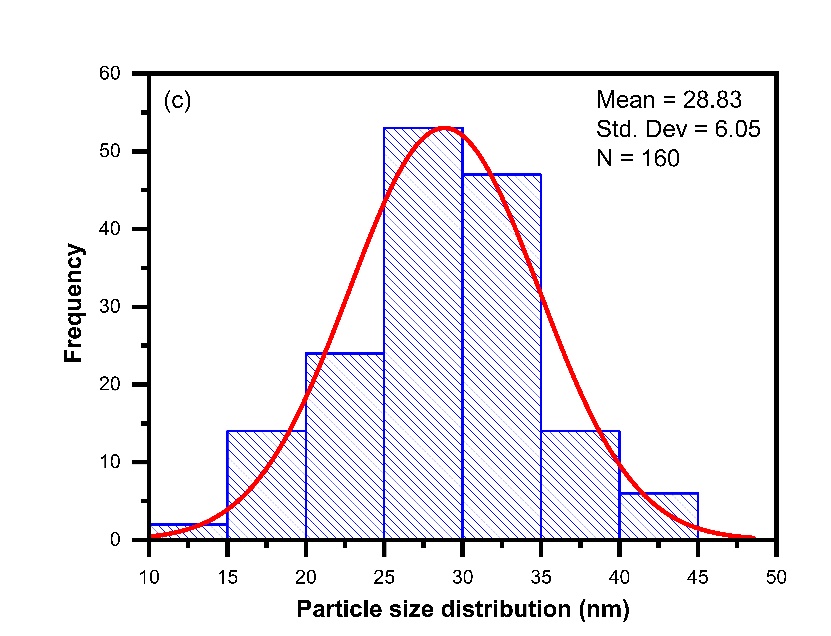


**Fig S1.** Particle size distribution of (a) pure ZnO, (b) ZnO/Chitosan nanocomposite and (c) MWCNTs/ZnO/Chitosan nanocomposite.
